# Single-Round Circular Aptamer Discovery Using Bioinspired Magnetosome-Like Magnetic Chain Cross-Linked Graphene Oxide

**DOI:** 10.34133/research.0372

**Published:** 2024-05-01

**Authors:** Lili Yao, Junmei Feng, Yuefei Zhou, Shengjie Gao, Shuai Liu, Hao Qu, Yu Mao, Lei Zheng

**Affiliations:** School of Food and Biological Engineering, Hefei University of Technology, Hefei 230009, China.

## Abstract

Circular aptamers are promising candidates for analytical and therapeutic applications due to their enhanced biological and structural stability. However, the process of circular aptamer selection remains a great challenge, as it requires multiple rounds of binding–separation–amplification that involves issues with nonspecific binding and amplification bias. Here, we develop a highly practical solution for reliable selection of circular aptamers in a single round based on magnetosome-like magnetic chain cross-linked graphene oxide (separation efficiency ≈ 10^5^). High-affinity aptamer candidates can be rapidly selected from a preenriched circular DNA library, while low-affinity candidates are effectively adsorbed and separated by magnetosome-like magnetic chain cross-linked graphene oxide. With lipopolysaccharide as a representative model, the single-round selected lipopolysaccharide circular aptamer has been identified to have a high binding affinity with a *K*_d_ value of low to nanomolar range. Using this method, circular aptamers for protein and small-molecule targets were also successfully generated. We envision that this approach will accelerate the discovery of various new circular aptamers and open up a new avenue for analytical and therapeutic studies.

## Introduction

Aptamers are single-stranded oligonucleotides, which can form unique conformations and bind to their targets with high affinity and selectivity [1,2]. They have been used in various analytical and therapeutic applications due to their advantageous features, such as compact size, small batch-to-batch variation, little or no immunogenicity, and low cost [3–9]. In recent years, circular aptamers have gained substantial interest because they possess an array of merits. First, circular aptamers exhibit enhanced biological stability as they are resistant to exonuclease degradation due to the lack of 5′ and 3′ ends, making them highly desirable for biological applications. Second, the circular form has increased the structural stability, ensuring them function well in complex matrix [10–14]. Furthermore, this relatively rigid structure makes circular aptamers have low entropy loss upon binding to their targets, thus increasing the chance of deriving high-affinity aptamers when using a circular nucleic acid library for selection [11,15–19]. Traditionally, aptamers are selected from the random-sequence oligonucleotide libraries via systematic evolution of ligands by exponential enrichment (SELEX) [20–22]. A typical SELEX procedure requires iterated rounds of binding–separation–amplification, until reaching the desired binder (target-binding oligonucleotides)-to-nonbinder (nonbinding oligonucleotides) ratio [23,24]. However, this multiround selection procedure is inherently prone to failure because of ineffective separation, polymerase chain reaction (PCR) amplification bias, and even conformational changes of the targets or aptamer candidates being selected [25,26]. An obvious solution to this daunting problem is increasing the separation efficiency (SE) to the level at which its single step becomes enough to reach the desired binder-to-nonbinder ratio [27–29].

Recently, the magnetic particles that modified graphene oxide (GO) (MGO)-based SELEX have garnered substantial attention owing to its simplified separation procedure compared to conventional GO-SELEX [30,31]. Moreover, this nonimmobilized selection approach preserves the natural binding states of the targets and aptamer candidates, thereby enhancing its reliability in practical applications. More importantly, this nonimmobilized approach can enable the selection of small molecules, ions, and other challenging targets that are difficult to immobilize [32,33]. However, the current synthesized magnetic graphene falls short in terms of achieving enough SE for a single round of selection.

**Fig. 1.**
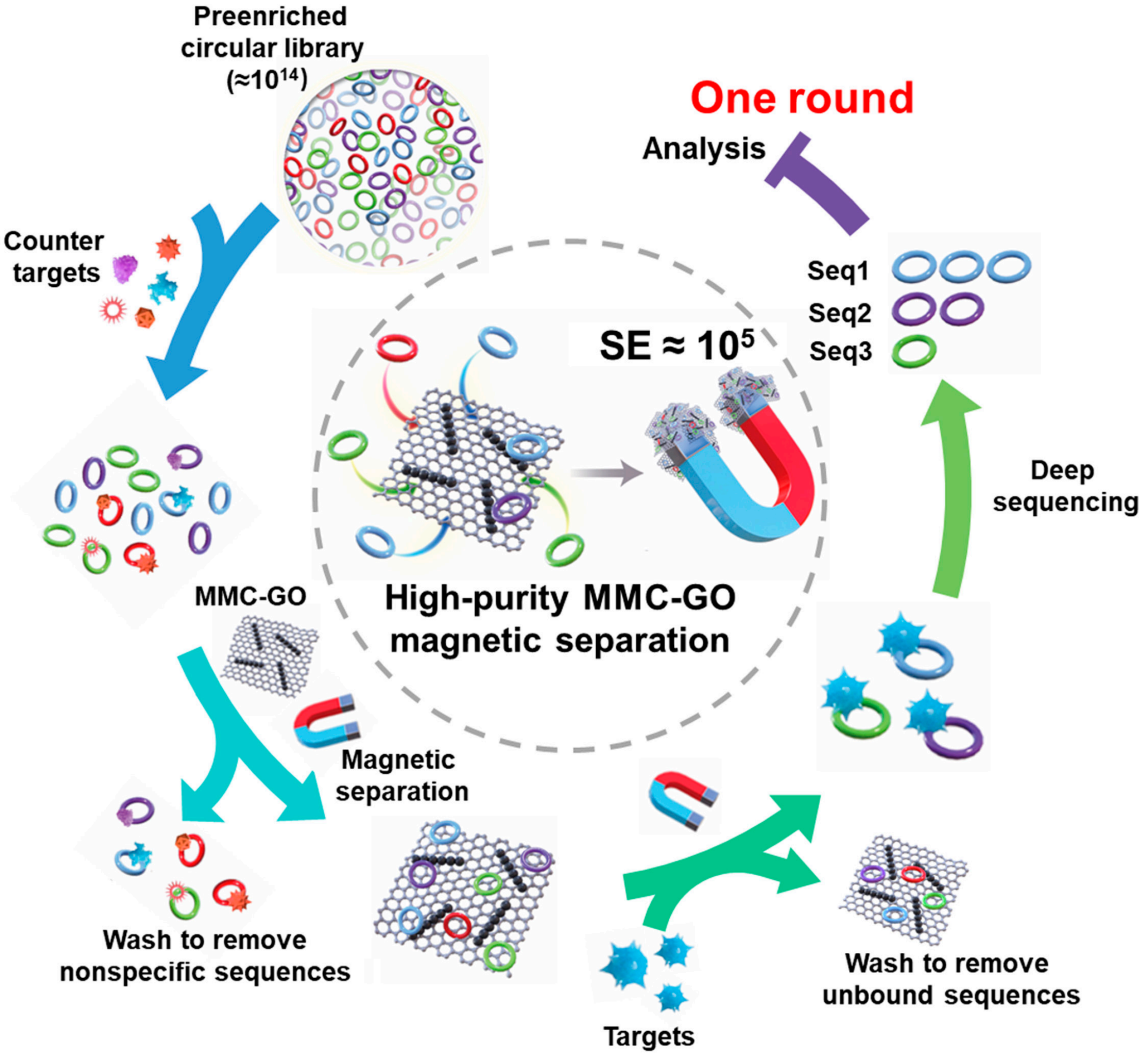
Schematic diagram of the MMC-GO-based single round of selection.

Inspired by the strong magnetic force exhibited by the magnetosome chain in magnetotactic bacteria (MTB), we biomimetically synthesized magnetosome-like magnetic chain cross-linked GO (MMC-GO). The prepared MMC-GO enables highly efficient separation of target sequences through a simple magnetic separation. The SE of MMC-GO-based method reaches 10^5^, which is hundreds to thousands of times higher than that of conventional methods. Consequently, the synthesized MMC-GO allows for effective and rapid adsorption and separation of free oligonucleotides, while high-affinity aptamer candidates are bound with targets and detached from MMC-GO. In addition, preenriched circular libraries (multiple copies) were used as initial libraries to augment the probability of discovering high-affinity aptamers [34]. Moreover, in light of recent advancements in high-throughput sequencing technologies, it is possible to evolve high-affinity aptamers from a preenriched circular library using high-purity (SE ≈ 10^5^) MMC-GO-based separation technology after a single round of selection (Fig. 1). With this optimized workflow, which encompasses preenriched circular libraries to improve the likelihood of finding high-affinity circular aptamers, counterselection to enhance sequence specificity, and high-purity MMC-GO separation technology to decrease the contamination of the target pool (Fig. 1), we have successfully obtained macromolecule [lipopolysaccharide (LPS)] circular aptamer with a dissociation constant (*K*_d_) value of low to nanomolar range using a single round of selection. Meanwhile, the selected aptamer exhibited excellent selectivity and biological stability. To validate the reliability of the proposed MMC-GO-based method, we also conducted single-round selection experiments using different types of targets, including protein [peanut allergen (Ara h1)] and small molecule [aflatoxin B_1_ (AFB_1_)], which resulted in the selection of high-affinity circular aptamers as well. Overall, we have established a single-round selection strategy, which can yield circular aptamers with high binding affinity, selectivity, and biological stability.

## Results

### Preparation of MMC-GO

**Fig. 2.**
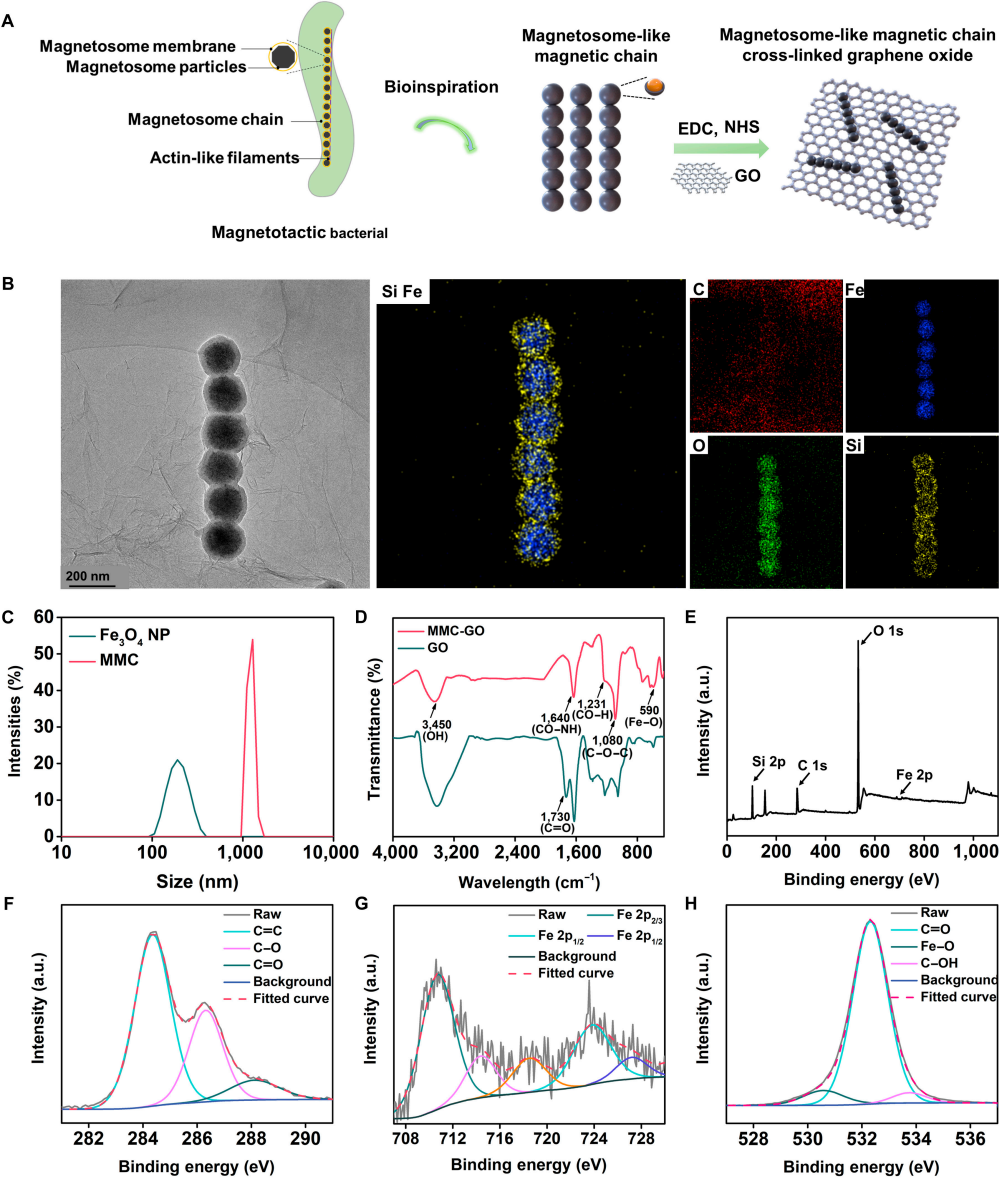
Characterization of the MMC-GO. (A) Schematic illustration of the synthesis process of MMC-GO. (B) TEM and the corresponding energy-dispersive spectrometer mapping images of MMC-GO. (C) Dynamic light scattering profiles of Fe_3_O_4_ NP and MMC. (D) Fourier transform infrared spectra of GO and MMC-GO. X-ray photoelectron spectroscopy spectra of (E) wide scan, (F) C 1s, (G) Fe 2p, and (H) O 1s of MMC-GO. a.u., arbitrary units.

Separation is a major challenge in SELEX [29,35]. To solve the problem of the target-bound single-stranded DNA (ssDNA) pool contamination with nonspecific binding sequences during separation, we fabricated the MMC-GO (Fig. 2A). As was shown in Fig. 2C and Fig. S1A and B, dynamic light scattering, transmission electron microscopy (TEM), and scanning electron microscopy revealed that numerous Fe_3_O_4_ nanoparticles (NPs) with an average diameter of 200 nm arranged into a chain-like structure, resembling the morphology of magnetosome chains in natural MTB, termed as MMC. They were randomly distributed and straight-like nanorods with an average size of 1.2 μm. This straight morphology could be attributed to the hardness of the coating layer SiO_2_, which was similar to the magnetosome membrane. The synthesized MMC-GO was characterized in detail. As was shown in Fig. S1C, the scanning electron microscopy revealed a substantial deposition of MMC onto the GO surface. The TEM images and the corresponding energy-dispersive spectrometer mapping images illustrated the existence of C, O, Fe, and Si elements with a homogeneous distribution in the MMC-GO, which helped improve the magnetic property and ssDNA adsorption capability of as-prepared materials (Fig. 2B). Meanwhile, the Fe elements focused on the core area, whereas Si elements concentrated on the shell area, forming the core–shell structures. The cross-linking of MMC-GO was further confirmed via Fourier transform infrared (Fig. 2D). In hybrids of MMC-GO, the peak at 1,730 cm^−1^ shifted to ~1,640 cm^−1^ was ascribed to the emergence of –COO^−^ after the reaction between –COOH on GO and –NH_2_ on MMC. The presence of a characteristic peak at 590 cm^−1^, attributed to the stretching vibration of the Fe–O bond in MMC, was observed in MMC-GO, suggesting that MMC was successfully conjugated onto the GO surface to form the MMC-GO hybrids. Other 3 typical characteristic peaks located at 3,450 cm^−1^ (–OH), 1,231 cm^−1^ (CO–H), and 1,080 cm^−1^ (C–O–C) could be clearly observed, which are assigned to basic-functional groups of GO [36]. X-ray photoelectron spectroscopy was used to analyze the surface chemical compositions and valence states of MMC-GO. Figure 2E showed the typical spectrum of wide scan, which confirmed that the MMC-GO contains C, O, Fe, and Si elements. The C 1s spectrum (Fig. 2F) could be fitted into 3 peaks of 284.6, 286.3, and 288.3 eV, which were assigned to C═C, C–O, and C═O bonds, respectively [37]. As shown in Fig. 2G, the peak of Fe 2p_3/2_ at 710.3 eV was ascribed to the presence of Fe_3_O_4_ [Fe(II) and Fe(III)] particles. Besides, the peak at 723.8 and 727.2 eV was attributed to Fe 2p_1/2_ [38]. Furthermore, the binding energies observed at 714.3 and 718.3 eV were designated to the satellite of Fe(II) and Fe(III), respectively. Three peaks were present in the O 1s spectrum of MMC-GO (Fig. 2H), which corresponds to Fe–O at 530.5 eV, C═O at 532.3 eV, and C–OH at 533.9 eV [37].

### DNA SE of the MMC-GO

**Fig. 3.**
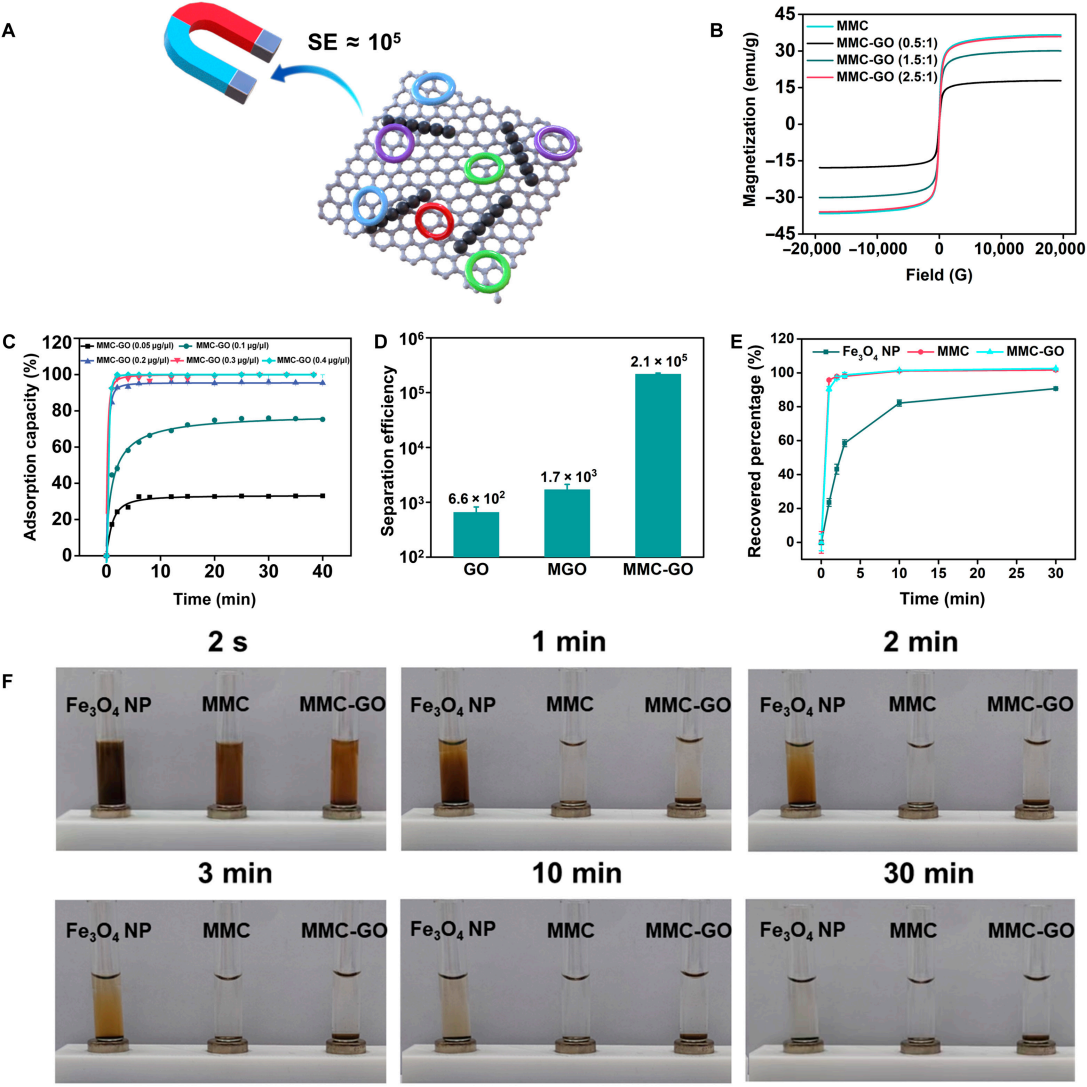
Magnetic separation performance of the MMC-GO. (A) Schematic illustration of SE based on MMC-GO. (B) Magnetization curves of MMC and MMC-GO with different loading amounts of magnetic chain. (C) DNA adsorption capacity as a function of the interaction time for MMC-GO of 0.05, 0.1, 0.2, 0.3, and 0.4 μg/μl. In each case, 10 pmol of ssDNA was used (the data represent the means ± SD, *n* = 3). (D) SE of the GO, MGO, and MMC-GO (the data represent the means ± SD, *n* = 3). (E) Recovered percentage (the data represent the means ± SD, *n* = 3) and (F) magnetic separation process of Fe_3_O_4_ NP, MMC, and MMC-GO with magnets at different separation times.

To enhance the SE of the fabricated MMC-GO, we optimized the coverage density of magnetic chains on MMC-GO. It was observed that the saturation magnetization values increased to 17.85, 30.08, and 35.89 emu/g, corresponding with the rising amounts of the magnetic chain being loaded (Fig. 3B). When the weight ratio of MMC:GO reached 2.5:1, MMC-GO exhibited a saturation magnetization similar to the magnetic chain, indicating that the MMC-GO could be manipulated by an external magnetic field and had no remanence when removing the magnetic field to prevent aggregation during the selection process. Moreover, to verify the feasibility of MMC-GO as a means of separation tool in a single round of selection, we tested the magnetic separation abilities of the optimized MMC-GO, MMC, and Fe_3_O_4_ NPs using magnets and analyzed the magnetic recovery efficiency at different times (Fig. 3E and F). It was worth noting that both prepared MMC and MMC-GO demonstrated significantly higher magnetic SE than Fe_3_O_4_ NPs, and the cross-linking with GO did not significantly affect the recovery capacity of the magnetic chains. Strikingly, more than 99% of the MMC and MMC-GO were rapidly and focally attracted to the magnet within 3 min, while only about 90% of Fe_3_O_4_ NPs were adsorbed until 30 min. Then, we studied the adsorption kinetics of MMC-GO for ssDNA (Fig. 3C). ssDNA (10 pmol) was completely adsorbed when the MMC-GO concentration reached 0.3 μg/μl. At the same time, MMC-GO reached its maximum adsorption rate of 120.23 ng/min (Fig. S2). Furthermore, we theoretically estimated the SE of the as-fabricated MMC-GO to ensure single round of selection [39]. In the MMC-GO-based single round of selection, SE was defined as the ratio of adsorbed circular ssDNA (circular ssDNA adsorbed by MMC-GO) and unadsorbed circular ssDNA (unadsorbed circular ssDNA separated from MMC-GO), when a certain amount of circular library was incubated with MMC-GO directly. Thus, large SE values indicated higher purity separation. Circular library (10 pmol) and MMC-GO (0.3 μg/μl) were incubated at room temperature (RT) for 20 min in 1× binding buffer. The unadsorbed circular ssDNA was quantified by rolling circle amplification (RCA). The measured SE of MMC-GO-based single round of selection was about (2.1 ± 0.1) × 10^5^, which significantly exceeded most conventional separation methods [SE (GO) ≈ (6.6 ± 1.6) × 10^2^ and SE (MGO) ≈ (1.7 ± 0.4) × 10^3^] (Fig. 3A and D and Fig. S3). Finally, to fully demonstrate the high efficacy of MMC-GO-based single round of selection, we conducted 2 parallel single round of selections using MGO and MMC-GO for direct comparison. The results revealed an increase in target binding capacity after both MGO-based and MMC-GO-based single round of selection. Notably, the pool obtained through MMC-GO-based single round of selection exhibited higher target binding capacity, demonstrating the superior efficacy of MMC-GO-based single round of selection (Fig. S4). Taken together, the highly efficient magnetic recovery capability conferred by MMC-GO and its high SE for ssDNA have laid a solid foundation for single round of selection.

### Selection of LPS circular aptamer based on a single round of selection

Following our assessment of MMC-GO performance, we investigated the feasibility of generating circular aptamer using MMC-GO-based single round of selection strategy. LPS was selected as the model target to test the feasibility of this strategy. As the key component of the Gram-negative bacterial outer membrane, LPS poses a substantial threat to public health by influencing various cellular and humoral-mediated systems [40]. Therefore, it is urgent to develop circular aptamers targeting LPS with high affinity, selectivity, and stability.

**Fig. 4.**
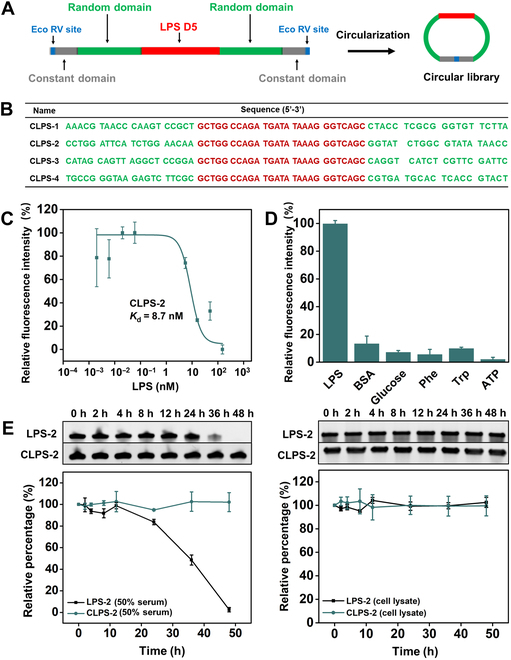
Characterization of the selected LPS circular aptamers. (A) The design of the LPS DNA library. (B) The sequence of top 4 LPS aptamer candidates. (C) Binding curve of CLPS-2 against LPS using MST assay (the data represent the means ± SD, *n* = 2). (D) Characteristic of the specificity of CLPS-2 using MMC-GO based binding assay (the data represent the means ± SD, *n* = 3). (E) Stability of CLPS-2 and LPS-2 in (left) 50% serum and (right) RAW 264.7 cell lysate (1,000 cells/μl) at 37 °C. Top: Gel images of CLPS-2 and LPS-2 after serum and cell lysate exposure for the indicated period of time. The fraction of intact CLPS-2 and LPS-2 (relative to the zero time point) was plotted as a function of time.

One of the best existing linear aptamers for LPS was subjected to truncation and optimization guided by its predicted secondary structure (Fig. S5) [41]. The tailored aptamer (LPS D5), which maintained high binding affinity and specificity (Fig. S6), was constructed as the seeding sequence for the circular library (Fig. 4A and Fig. S7A). First, the circular library was amplified (50 copies of each sequence), which was termed preenriched circular library. Subsequently, biological complex medium (human serum) was mixed with the preenriched circular library in the counterselection step to enhance the selectivity and sensitivity of sequences (Fig. S7B). Nonbinding sequences were adsorbed and separated using MMC-GO. The target was introduced to bind with a candidate circular DNA pool. Finally, target binding sequences were separated and sequenced using a previously reported method (Fig. S7C) [11].

### Characterization of the selected LPS circular aptamers

The top 4 ranked candidate sequences from deep sequencing were chosen for binding performance characteristics using MMC-GO-based binding assay (Fig. 4B). Each circular aptamer was incubated with LPS, and the bound sequences were isolated and analyzed by fluorescence spectrum. Among the 4 candidates, CLPS-2 exhibited strong binding activity toward LPS (Fig. S8). Subsequently, its binding affinity and specificity were determined using MMC-GO-based binding assay. Notably, CLPS-2 (Fig. S9) exhibited a high target binding ability, as evidenced by a *K*_d_ value down to 2.9 nM, coupled with remarkable specificity (Fig. 4D and Fig. S10). In addition, microscale thermophoresis (MST) assay was applied to further determine the affinity of CLPS-2, which yielded a similar *K*_d_ value (*K*_d_ ≈ 8.7 nM; Fig. 4C). In contrast, the linear counterpart of CLPS-2 (LPS-2) was unable to bind with LPS (Fig. S11). We speculate that there are 2 main reasons for this result. One reason is that the linear counterpart may exhibit a different topology that cannot recognize the target molecule. Another reason is that the circular aptamer possesses enhanced thermal stability. As was shown in Table S2, CLPS-2 exhibited higher thermal melting temperature (*T*_m_ value) than LPS-2, providing a more stable overall structure for target binding, thus achieving higher affinity and selectivity. Moreover, it is worth noting that while the circular aptamer can resist exonuclease activity, it may be susceptible to endonuclease in complex biofluid. The NEBcutter was used to identify the potential endonuclease recognition sites within CLPS-2. It was revealed that several potential endonuclease recognition sites were presented in CLPS-2 (Fig. S12). Therefore, we investigated the biostability of CLPS-2 and its linear counterpart (LPS-2) in human serum. The circular form, CLPS-2, was resistant to exonuclease degradation, maintaining its sequence integrity even after a 48-h exposure to 50% human serum. In contrast, LPS-2 underwent rapid degradation, with a noticeable breakdown occurring after 12-h incubation in 50% human serum (Fig. 4E, left). We further analyzed the stability of CLPS-2 in RAW 264.7 cell lysate (1,000 cells/μl). It was found that CLPS-2 remained stable even after 48 h in the cell lysate. Meanwhile, LPS-2 also remained stable after incubation in cell lysate for 48 h (Fig. 4E, right). It is possible that the relatively low concentrations of both exonuclease and endonuclease in the cell lysate are insufficient to degrade the linear counterpart [12,42,43]. Moreover, the storage stability of CLPS-2 was also tested. It was shown that CLPS-2 remained stable for even 30 d at RT. While LPS-2 can only be kept for 3 d (Fig. S13). The excellent biostability of circular aptamers makes them highly desirable for applications in authentic biological samples without a temperature-controlled supply chain. Overall, these results indicated the fact that the high-quality circular aptamers targeting macromolecular polymer LPS can be successfully obtained on the basis of the proposed single-round selection strategy.

### Selection of Ara h1 and AFB_1_ circular aptamers based on single round of selection

**Fig. 5.**
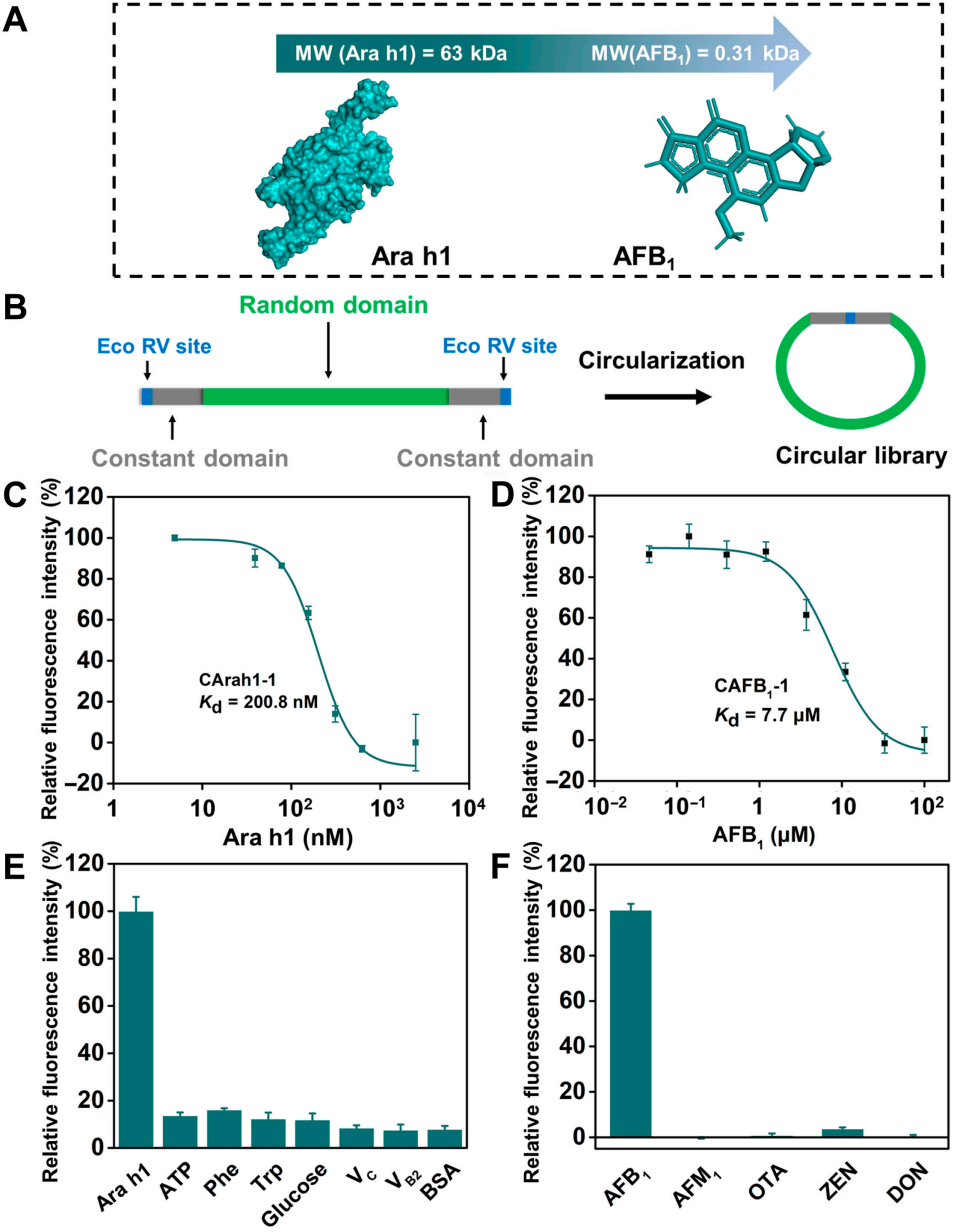
Characterization of the selected Ara h1 and AFB_1_ circular aptamers. (A) Structure basis of Ara h1 and AFB_1_. MW, molecular weight. (B) The design of the Ara h1 and AFB_1_ DNA libraries. (C) Binding curve of CArah1-1 against Ara h1 using MST (the data represent the means ± SD, *n* = 2). (D) Binding curve of CAFB_1_-1 against AFB_1_ using MST (the data represent the means ± SD, *n* = 2). (E) Characteristic of the specificity of CArah1-1 using MMC-GO-based binding assay (the data represent the means ± SD, *n* = 2). (F) Characteristic of the specificity of CAFB_1_-1 using MMC-GO-based binding assay (the data represent the means ± SD, *n* = 3).

To faithfully demonstrate the broad applicability of the proposed MMC-GO-based single round of selection as a versatile technology platform for diverse targets, we expanded the targets range to include protein (Ara h1, one of the major peanut allergens) and even small molecule (AFB_1_, an extremely toxic aflatoxin) (Fig. 5A) [44,45].

In Ara h1 single round of selection, we used a completely random circular library except for the constant primer region (Fig. 5B and Fig. S14A). First, peanut crude proteins, serving as counterselection targets, were incubated with a preenriched circular DNA library to enhance the specificity of the selected sequences (Fig. S14B) [46]. Subsequently, MMC-GO was utilized to adsorb nonbinding circular sequences, which was followed by the introduction of the target to bind with the candidate circular DNA pool. After that, target binding sequences were isolated and sequenced, from which a few candidate sequences were further tested. Among the 5 candidates, CArah1-1, CArah1-3, and CArah1-4 demonstrated notable binding activity toward Ara h1, and their binding affinities were then assessed using MMC-GO-based fluorescent detection assay (Figs. S14C and S15A). Notably, CArah1-1 demonstrated a high target binding ability with a *K*_d_ value of 980.7 nM (Figs. S15B to D and S17A), which is comparable to that of previously published Ara h1 aptamer [80 nucleotides (nt); *K*_d_ ≈ 1.2 μM] [44] obtained through multiple rounds of selection (Fig. S16A). MST assay was further used to determine the binding affinity of CArah1-1, showing a *K*_d_ value of 200.8 nM (Fig. 5C). Furthermore, we validated the binding specificity of the representative circular aptamer CArah1-1 and previously published Ara h1 aptamer against some latent interfering substance in food, such as bovine serum albumin, glucose, phenylalanine (Phe), tryptophan (Trp), Vitamin C (V_C_), Vitamin C (V_B2_), and adenosine triphosphate (ATP). As shown in Fig. 5E and Fig. S16B, CArah1-1 demonstrated slightly enhanced specific binding toward Ara h1 and almost no interaction with other interferents.

The aptamer selection against AFB_1_ was consistent with that against LPS and Ara h1. To enhance specificity, we used several prevalent mycotoxins, including aflatoxin M_1_ (AFM_1_), ochratoxin A (OTA), zearalenone (ZEN), and deoxynivalenol (DON), as counterselection targets (Fig. S18B). The random circular library (Fig. 5B and Fig. S18A) was amplified and incubated with interfering mycotoxins. MMC-GO was then introduced to the mixture for the separation of unbound sequences. Subsequently, AFB_1_ was added and incubated with the nonbinding circular sequences. The sequences bound to AFB_1_ were sequenced, and the candidate sequence CAFB_1_-1 was further evaluated using the same MMC-GO-based binding assay (Figs. S18C and S19). CAFB_1_-1 had a *K*_d_ value of 8.6 μM (Fig. S20), which is similar to a previously published AFB_1_ aptamer (50 nt; *K*_d_ ≈ 8.1 μM) [45] evolved from multiple rounds of selection (Fig. S21A). Another binding test, MST assay, was applied to confirm the affinity of the CAFB_1_-1, showing a *K*_d_ value of 7.7 μM (Fig. 5D). The selectivity of CAFB_1_-1 and a previously published AFB_1_ aptamer were also investigated by examining their binding abilities toward 4 other different toxins. Notably, they all displayed apparent specifically toward AFB_1_ (Fig. 5F and Fig. S21B).

In summary, the effective isolation of circular aptamers targeting Ara h1 and AFB_1_ demonstrates that the MMC-GO-based single-round selection strategy is effective for discovering aptamers against diverse targets, including macromolecular proteins, as well as small molecules that are lacking functional moieties.

## Discussion

SELEX is a progressive process that involves repeated cycles of binding–separation–amplification to refine the library and identify sequences with high affinity for the target molecule. Therefore, ensuring minimal nonspecific binding during the separation phase is crucial for aptamer enrichment, highlighting the pivotal role of high-purity separation technologies in enhancing selection efficiency and discovering high-affinity sequences.

As a natural magnetic NPs of biological origin, the magnetosome was a special “organelle” formed by biomineralization in MTB. These ferrimagnetic NPs of high saturation magnetization and with high coercive fields were enveloped by membranes and generally organized into chains, which was essential for MTB magnetic navigation response to geomagnetic fields [47]. Inspired by the strong magnetic force exhibited by the magnetosome chain in MTB, we hypothesized that cross-linking the synthesized MMC onto the surface of GO could endow GO with better magnetic manipulation ability, thereby eliminating the contamination of the target-bound ssDNA pool during separation. In the proposed selection strategy, we developed a high-purity separation technology [SE ≈ (2.1 ± 0.1) × 10^5^] based on MMC-GO, which significantly exceeded most conventional separation methods [SE (GO) ≈ (6.6 ± 1.6) × 10^2^ and SE (MGO) ≈ (1.7 ± 0.4) × 10^3^]. This was attributed to the enhanced collective magnetic property conferred by MMC. Magnetic chains often exhibit higher magnetic anisotropy compared to dispersed NPs, which favors the coalignment of magnetic moments in one direction. [48,49]. In addition, under the magnetic field, compared with the dispersed magnetic NPs, the chain-like arrangement of NPs in the magnetic chains exhibited stronger dipole interactions, resulting in higher magnetic separation capacity [50,51]. Consequently, the synthesized MMC-GO exhibited superior capabilities for the rapid adsorption and subsequent separation of free oligonucleotides. Conversely, high-affinity aptamer candidates are bound with targets and dissociated from MMC-GO. In addition, preenriched circular libraries were used as initial libraries to augment the probability of finding high-affinity aptamers. Conventionally, a typical SELEX starts with a random library containing about 10^13^ to 10^14^ DNA or RNA sequences. It appears that higher-affinity sequences are much less common in the library than low-affinity sequences. Consequently, the likelihood of very high-affinity sequences being present in the initial library may be diminished.

With the MMC-GO-based single-round selection, we have isolated circular aptamers against a variety of targets, including the macromolecular (LPS), protein (Ara h1), and small molecular (AFB_1_), which exhibit 3 outstanding properties: high binding affinity, high selectivity, and high biological stability. Furthermore, cyclization can improve the conformational stability of oligonucleotides, thus increasing their thermal stability, which is expected to provide a more stable overall structure for target binding. Despite AFB_1_ being the smallest molecule among our targets, no particular advantages or disadvantages were noted in the enrichment of the aptamer pool for AFB_1_ compared to the other 2 macromolecules. However, it is worth noting that the selection against AFB_1_ did not exhibit an equivalent level of excellent affinity as observed with large molecular targets, which may be attributed to several factors inherent to small molecular, including its reduced accessible surface area, the limited number of available chemical moieties, and its simpler structural complexity relative to larger species [52]. It is conceivable that the affinity of aptamers for small molecules may be enhanced through multiple rounds of incubation and separation. In addition, while preenriched circular libraries increase the possibility of discovering high-affinity aptamers, they also inevitably reduce library diversity to some extent. Therefore, further investigation into methods for identifying high-affinity sequences from more diverse libraries is warranted.

In summary, we have demonstrated a promising strategy toward the simple, rapid, and universal generation of circular DNA aptamers. Unlike conventional SELEX methods, our approach can isolate circular aptamers in a single round of selection. The successful isolation of circular aptamers with single round of selection highlights its advantages in discovering high-affinity aptamers with high efficiency and, more importantly, producing high-quality recognition elements with improved chemical and functional stabilities. Overall, our findings demonstrate the feasibility and potential benefits offered by MMC-GO-based single round of selection to promote efficiency in discovering circular aptamers. We envisage that the strategy presented in this study will open up new opportunities for future exploitation of circular aptamers in analytical and therapeutic applications.

## Materials and Methods

### Experimental design

The primary objective of this study was to develop a method for efficient selection of circular aptamers in a single round. An MMC-GO has been synthesized and enables highly efficient separation of the candidate oligonucleotides with an SE of 10^5^, which is hundreds to thousands of times higher than that of conventional methods. Using this method, we have successfully isolated circular aptamers against a variety of targets, including macromolecular (LPS), protein (Ara h1), and even small molecular (AFB_1_), which exhibit high binding affinity, excellent selectivity, and good biological stability.

### MMC-GO preparation

The synthesis of GO was as follows. The GO was prepared using the modified Hummer’s method. The graphite (0.5 g) was added slowly into 18 ml of concentrated H_2_SO_4_ and stirred 30 min. Then, 6 ml of concentrated HNO_3_ was introduced. Next, 2.5 g of KMnO_4_ was added, accompanied by vigorous stirring 2 h while keeping the temperature below 20 °C. The reaction was terminated by the introduction of 60 ml of additional deionized water and 3 ml of 30% H_2_O_2_ solution sequentially. The filtered product was washed several times with water until the pH reached neutral and finally washed with ethanol and vacuum dried at 50 °C.

The synthesis of Fe_3_O_4_ NPs was as follows. First, 0.55 g of FeCl_3_·6H_2_O and 1.5 g of sodium acetate were added to 20 ml of ethylene glycol and violently mixed for 30 min to get a dark-brown suspension. The above suspension was transferred to a Teflon-sealed autoclave and reacted under 200 °C for 12 h. The resulting product was washed 3 times with anhydrous ethanol and deionized water, followed by vacuum drying at 50 °C.

The synthesis of MMC was as follows. The product Fe_3_O_4_ NPs were introduced to 60 ml of 95% alcohol and ultrasonicated for 15 min. One milliliter of ammonium hydroxide was added into the system and ultrasonicated for another 15 min. Then, 0.3 ml of tetraethyl orthosilicate was added and ultrasonicated for 15 min. After stirred for 2 h, the obtained product was washed 3 times with anhydrous ethanol and deionized water, followed by dissolved in 1 ml of ethanol. After that, the product was dispersed in 60 ml of 95% alcohol and ultrasonicated for 15 min. After pH adjusted to 5.0, 1.2 ml of 3-aminopropyltriethoxysilane was introduced and incubated for 2 h. The obtained product was washed 3 times with anhydrous ethanol and deionized water using magnetic separation, followed by dissolving in 3 ml of deionized water.

The synthesis of MMC-GO was as follows. Briefly, GO (1 mg/ml) was ultrasonicated for 30 min. Subsequently, to facilitate the activation of carboxyl groups on GO surface, 550 μl of GO suspension was incubated with 250 μl of 1-ethyl-3-(3-dimethylaminopropyl)carbodiimide (EDC) (0.1 M) and 120 μl of *N*-hydroxysuccinimide (NHS) (0.1 M) in 0.25 M MES buffer (pH 5.0) at 37 °C for 1 h, Then, 100 μl of amino-functionalized magnetic chains (15 mg/ml) were introduced to the above mixture and incubated at 37 °C overnight. Finally, the resulting amino-functionalized MMC-GO was washed 3 times with 1× PBSMT (pH 7.2, containing 137 mM NaCl, 2.68 mM KCl, 8.1 mM Na_2_HPO_4_, 1.76 mM KH_2_PO_4_, 1 mM MgCl_2_, and 0.025% Tween 20) and redispersed in 200 μl of 1× PBSMT.

### Adsorption kinetics of MMC-GO for ssDNA

Circular ssDNA (10 pmol) was dissolved in 1× PBSMT, heated at 95 °C for 5 min, and then cooled on ice for 15 min. Different amounts of MMC-GO were introduced to the above solution. Next, the mixture was incubated at RT for different times and placed on magnetic test tube holder. Obtained flow-through was collected, mixed with 1× SYBR Gold, and measured by fluorescent spectroscopy (*F*_t_). The initial fluorescent intensity is *F*_0_. With time, this adsorption increases and reaches a saturation maximum. Adsorption (%) = (*F*_0_ − *F*_t_) / *F*_0_ × 100%.

### Measurement of the SE

In the single round of selection, we defined SE as the ratio between the amount of adsorbed circular ssDNA (circular ssDNA adsorbed by GO, MGO, and MMC-GO) and the unadsorbed circular ssDNA (unadsorbed circular ssDNA separated from GO, MGO, and MMC-GO), when a certain amount of circular library was incubated with GO, MGO, and MMC-GO, respectively. First, the relationship between the concentrations of circular library and their corresponding fluorescence intensity were measured to establish a calibration curve for SE calculation. Specifically, RCA was used to amplify the signal since the concentrations of circular library were too low to be detected directly. Therefore, the various concentrations of the circular library were subjected to RCA and subsequently incubated with SYBR Gold, the ssDNA binding fluorescent dye, to establish a calibration curve. Subsequently, 10 pmol of circular library was incubated with certain amounts of GO, MGO, and MMC-GO solutions at RT for 20 min, respectively. The mixture was then subjected to magnetic separation. The supernatant (unadsorbed circular ssDNA) was collected and amplified by RCA to derive a relative fluorescence signal. The amount of the adsorbed circular ssDNA was calculated by the difference between the total amount of circular library and the amount of unadsorbed circular ssDNA. The fluorescence signal was determined by fluorescence spectrum ( λ_ex_/λ_em_ = 498/540 nm).

### Characterization of the truncated LPS aptamers

LPS aptamer, which was previously selected according to nonequilibrium capillary electrophoresis of equilibrium mixtures experiments [41]. Aptamer was tailored guided by its predicted secondary structure (Fig. S5). Overall, 5 truncated aptamers sequences (LPS D1 to D5) were obtained. Then, 10 pmol of FAM-labeled LPS sequence and its truncated counterparts were incubated with various concentrations of LPS in 1× PBSMT for 1 h. Then, MMC-GO was introduced and the mixture was incubated at RT for 20 min. Following magnetic separation, the fluorescence intensity of the target-bound aptamer was measured at 485 nm. The fluorescence intensity was measured without LPS in the control group. The recorded fluorescence data were normalized and subsequently subjected to curve fitting using the *K*_d_ fit equation.

### Preparation of peanut crude protein

The preparation of peanut crude protein was reported by Roberts’ group [46] previously. Briefly, a mixture of raw peanuts was prepared, and their seed coats were eliminated prior to homogenization. The resulting homogenate underwent defatting using *n*-hexane, followed by extraction of crude protein through vigorous vertexing the mixture containing 45 mg of peanut flour in 20 mM tris buffer (pH 8.5). This mixture was then incubated on a shaker at 21 °C for 30 min. Subsequently, the aqueous layer was harvested by centrifugation at RT and a speed of 12,600*g*.

### Library preparation

The preparation of preenriched circular library was as follows. The LPS DNA library contained a 27-nt LPS binding truncated sequence flanked by 2 random domains of 20 nt and 2 constant domains at each of the 5′ and 3′ end (Fig. S7A). The AFB_1_ and Ara h1 library was 60 nt long, consisting of a 40-nt random domain flanked by 2 constant domains of 10 nt, as shown in Figs. S14A and S18A. Notably, the primer domains contained a distinct cleavage site for the Eco RV. First, the phosphorylation of linear DNA oligonucleotide was reacted in 60 μl of 1× PNK buffer [50 mM tris-HCl (pH 7.6), 10 mM MgCl_2_, 5 mM dithiothreitol (DTT), and 0.1 mM spermidine] containing 1.5 nmol of linear oligonucleotide, 1 mM ATP, and 20 U of PNK. The reaction is incubated at 37 °C for 40 min and then heated at 90 °C for 5 min. Subsequently, 306 μl of H_2_O and 2 μl of DNA template (LT1 or LT2; 100 μM) were added to facilitate the circularization. The reaction mixture was heated at 90 °C for 3 min and cooled down to RT for 10 min. After that, T4 DNA ligase buffer [40 mM tris-HCl, 10 mM MgCl_2_, 10 mM DTT, and 0.5 mM ATP (pH 7.8) at 25 °C] and 2 μl of T4 DNA ligase (5 U/μl) were introduced. After reacting at RT for 3 h, the mixture was incubated at 90 °C for 5 min. Finally, the ligated circular DNA pools underwent ethanol precipitation and were then purified by 10% denaturing polyacrylamide gel electrophoresis (dPAGE). Quantification of the DNA concentration was ascertained by the absorption spectrum.

The preparation of preenriched circular library was as follows. The circular ssDNA library (10 pmol of random DNA molecules) underwent 2 rounds of RCA, followed by Eco RV restrict digestion and DNA ligation for circulation. The RCA was typically conducted in 50 μl of 1× RCA buffer [comprising 33 mM tris-acetate (pH 7.9) at 37 °C, 10 mM magnesium acetate, 66 mM potassium acetate, 0.1% (v/v) Tween 20, and 1 mM DTT] containing the circular DNA library (10 pmol), 2 μl of LT1 (10 pmol), and 1 mM deoxyribonucleoside triphosphate (dNTP). Subsequently, the above mixture was heated at 90 °C for 3 min and cooled down to RT for 10 min. Then, 0.5 μl of phi29 DNA polymerase (10 U/μl) was introduced and incubated at 30 °C for 60 min before being heated at 90 °C for 5 min to deactivate the polymerase. Following this step, 2 μl of 50 μM LT2 was introduced to the above mixture. The resulting mixture was heated at 90 °C for 5 min and cooled down to RT for 10 min. Next, 10 μl of 10× fast digestion buffer [100 mM tris-HCl (pH 8.0), 50 mM MgCl_2_, 1 M NaCl, and bovine serum albumin (1 mg/ml)] and 5 μl of fast digest Eco RV (2 U/μl) were added into above mixture, and the final total reaction volume was 100 μl. The above mixture was incubated at 37 °C for 8 h and heated at 90 °C for 5 min to inactivate the restriction enzyme. Finally, the amplified monomerized RCA products were ligated into circular DNA template B and purified by 10% dPAGE. The circular DNA template B was eluted from the gel for the second round of RCA. The reaction conditions were the same as in the first RCA except that LT1 is replaced by LT2. For the restriction enzyme digestion process, LT1 was used instead of LT2. Finally, the preenriched circular DNA library was purified by 10% dPAGE for subsequent single round of selection.

### Characteristic the efficiency of single round of selection

Fifty-fold preenriched circular DNA library (500 pmol) was heated at 95 °C for 5 min, then cooled on ice for 15 min, and incubated with 20% serum in 100 μl of 1× PBSMT at RT for 1 h. Next, MGO (25 μg/μl) and MMC-GO (15 μg/μl) were introduced. Following the incubation at RT for 20 min, the tube was placed on a magnetic test tube holder, allowing for the separation and subsequent removal of the supernatant. The MGO and MMC-GO were washed 3 times with 1× PBSMT. The isolated MGO and MMC-GO were then incubated with LPS (50 nM) in 100 μl of 1× PBSMT at RT for 20 min. After that, the mixture was placed on a magnetic test tube holder, and the supernatant was collected. Finally, the pools from MGO-based and MMC-GO-based single round of selection were amplified. The preenriched circular library was used as the initial library. The relative LPS binding capacity of the different pools was analyzed by the MMC-GO-based binding assay.

### Single round of selection

For the LPS single round of selection, a 50-fold preenriched circular DNA library (500 pmol) was heated at 95 °C for 5 min, then cooled on ice for 15 min, and incubated with 20% serum in 100 μl of 1× PBSMT at RT for 1 h. Next, MMC-GO (15 μg/μl) was introduced. Following the incubation at RT for 20 min, the tube was placed on a magnetic test tube holder, allowing for the separation and subsequent removal of the supernatant. The MMC-GO was washed 3 times with 1× PBSMT. The isolated MMC-GO was then incubated with LPS (50 nM) in 100 μl of 1× PBSMT at RT for 20 min. Finally, the mixture was placed on a magnetic test tube holder, and the supernatant was collected.

The single round of selection for AFB_1_ and Ara h1 was similar to LPS, except that the counter MMC-GO selection was replaced by different toxins (ZEN, OTA, AFM_1_, and DON) and peanut crude protein.

### Sequencing protocol

The eluted DNA, after single round of selection, was digested, diluted at different times, and amplified by PCR step. In PCR, a 50-μl reaction mixture was prepared containing the diluted DNA (1 μl), forward primer (F1; 0.4 μM) and reverse primer (F2; 0.4 μM), 1× PCR buffer [75 mM tris-HCl (pH 9.0), 2 mM MgCl_2_, 50 mM KCl, 20 mM (NH_4_)_2_SO_4_, and 200 μM dNTP], and 1.5 U of Taq DNA polymerase. The DNA amplification was carried out under the following thermal cycler conditions: 94 °C for 3 min; 13 cycles at 94 °C (30 s), 52 °C (45 s), and 72 °C (45 s); and 72 °C for 1 min. The obtained DNA underwent ethanol precipitation and was subsequently analyzed using high-throughput sequencing by Sangon Biotech Company.

### MMC-GO-based binding assay

To characterize the binding affinity and selectivity of the isolated aptamers. Each aptamer candidate and library sequence were heated at 95 °C for 5 min, then cooled on ice for 15 min, and incubated with the target in 1× binding buffer. After incubation for 60 min, the MMC-GO solution was added. After 20 min of incubation, the tube was placed on a magnetic test tube holder, and the supernatant remained. The circular aptamer bound with the target was incubated with the fluorescent dye SYBR Gold, and the fluorescence signal was determined by fluorescence spectrum (λ_ex_/λ_em_ = 498/540 nm).

### Microscale thermophoresis

Ara h1 proteins were labeled using Monolith RED-HNS second-generation protein labeling kit. MST binding assays were conducted using a fixed concentration of fluorescently labeled Ara h1 in 1× binding buffer and various concentrations of aptamers. The assays were performed utilizing a Monolith NT.115 Pico (NanoTemper Technologies, Munich, Germany) with the temperature controlled at 25 °C, using standard capillaries for sample test. The recorded fluorescence data were normalized and subsequently subjected to curve fitting using the *K*_d_ fit equation. In addition, MST binding assays for LPS were conducted using a fixed concentration of fluorescein-isothiocyanate-labeled LPS in 1× binding buffer and various concentrations of aptamers. Furthermore, MST binding assays for AFB_1_ were conducted using a fixed concentration of fluorescently labeled CAFB_1_-1 aptamers in 1× binding buffer and various concentrations of AFB_1_.

### Thermal melting temperature analysis

Melting profiles were acquired and analyzed on a QuantStudio 5 real-time PCR detection system (Thermo Fisher Scientific). Five micromolars of CLPS-2 and LPS-2 were incubated with 1× TB Green in 1× binding buffer at 16 °C for 30 min and then slowly heated to 95 °C with a heating rate of 0.5 °C/30 s to acquire a fluorescence signal.

### Stability assay

The circular aptamer (6 μM) and its linear variant were subjected to incubation in 50% human serum at the temperature of 37 °C for 0, 2, 4, 8, 12, 24, 36, and 48 h. At the determined point, samples were subjected to heat inactivation at 95 °C for 10 min and preserved at 20 °C for further analysis.

The circular aptamer (6 μM) and its linear variant were subjected to incubation at RT for 0, 1, 3, 5, 7, 14, 21, and 30 d. At the determined point, samples were subjected to heat inactivation at 95 °C for 10 min and preserved at 20 °C for further analysis.

The circular aptamer (6 μM) and its linear variant were incubated with RAW 264.7 cell lysate (1,000 cells/μl) at the temperature of 37 °C for 0, 2, 4, 8, 12, 24, 36, and 48 h. At the determined point, samples were subjected to heat inactivation at 95 °C for 10 min and preserved at 20 °C for further analysis.

The above samples were subsequently combined with 2× denaturing gel loading buffer and then subjected to analyze by 10% dPAGE.

### Statistical analysis

All data were analyzed by calculating averages and SDs.

## Data Availability

All data needed to evaluate the conclusions in the paper are present in the paper and the Supplementary Materials.
