# Supplementary material for: Single-Round Circular Aptamer Discovery Using Bioinspired Magnetosome-Like Magnetic Chain Cross-Linked Graphene Oxide
Source: Research (Wash D C). 2024 May 1;7:0372. doi: 10.34133/research.0372 (PMC11062507; doi:10.34133/research.0372)
Supplement: Supplementary 1 — Supplementary Text Figs. S1 to S21 Table S1 and S2 [file research.0372.f1.zip › RESEARCH_SI-Clean-20240401.docx]

**Supplementary Materials**

**Single-Round Circular Aptamer Discovery**

Lili Yao,^1,†^ Junmei Feng,^1,†^ Yuefei Zhou,^1^ Shengjie Gao,^1^ Shuai Liu,^1^ Hao Qu,^1^ Yu Mao,^1,^* and Lei Zheng^1,^*

* Corresponding author. Email: maoyu@hfut.edu.cn; lzheng@hfut.edu.cn

**This PDF file includes:**

Supplementary Text

Fig. S1 to S21

Table S1 to S2

Supplementary Text

Oligonucleotides and other materials

All DNA oligonucleotides (Supplementary Table S1) used in this work were synthesized and purified with HPLC in Sangon Biotech Company (Shanghai, China). Recombinant lipopolysaccharide (LPS, from E.coli O55:B5), 1-ethyl-3-(3-dimethyl aminopropyl) carbodiimide (EDC), N-hydroxysuccinimide (NHS), Zearalenone (ZEN, from Giberella zeae), Deoxynivalenol (DON, from Fusarium niveum) and ochratoxin A (OTA, from Aspergilus ochraceus) were purchased from Sigma Aldrich (St. Louis, MO, USA); Aflatoxferric chloride hexahydrate (FeCl_3_·6H_2_O), sodium acetate, tetraethyl orthosilicate (TEOS), (3-aminopropyl) triethoxysilane (APTES), glucose, bovine serum albumin (BSA), Aflatoxin B_1_ (AFB_1_, from Aspergillus flavus), Vitamin C (V_C_), Vitamin B_2_ (V_B2_), phenylalanine (Phe), tryptophan (Trp) and 4S Red Plus were purchased from Sangon Biotech Company (Shanghai, China); T4 polynucleotide kinase (PNK), T4 DNA ligase, phi29 DNA polymerase, EcoRV, adenosine 5′-triphosphates (ATP) and deoxyribonucleoside 5′-triphosphates (dNTPs) were purchased from New England Biolabs (NEB, Beijing, China); Aflatoxin M_1_ (AFM_1_, from Aspergillus flavus) was purchased from J&K Scientific (Beijing, China); Ara h1 was purchased from Anti Biotechnology (Shenzhen, China); human serum was purchased from XinFan Biotech (Shanghai, China); graphite powder was purchased from XFNANO Materials Tech. Co. (Nanjing, China); SYBR^TM^ Gold was purchased from Thermo Fisher (Waltham, USA). Water was purified with a Milli-Q Synthesis from a Millipore system.

Instruments

The fluorescent images of gels were obtained using Tanon MINI Space System. The intensity of each band was analyzed using ImageJ software. Fluorescence measurements were carried out in a 96-well assay plate using a microplate reader (THERMO Varioskan Flash).


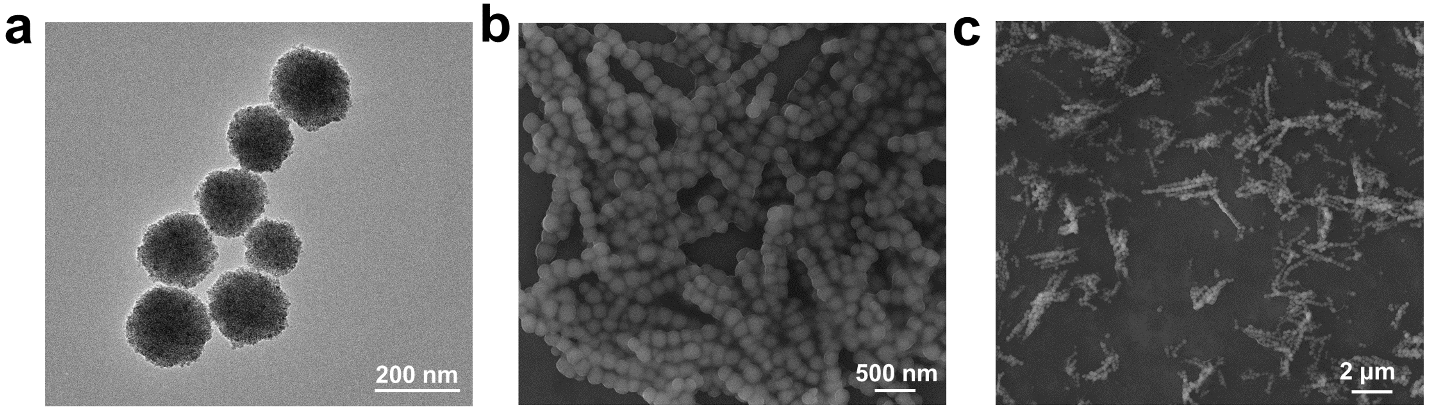


Fig.S1. Characterization of the Fe_3_O_4_ NP, MMC and MMC-GO. a) TEM of Fe_3_O_4_ nanoparticles. SEM of b) MMC and c) MMC-GO.





Fig.S2. Adsorption rate of MMC-GO (The data represent the mean ± SD, n=3).


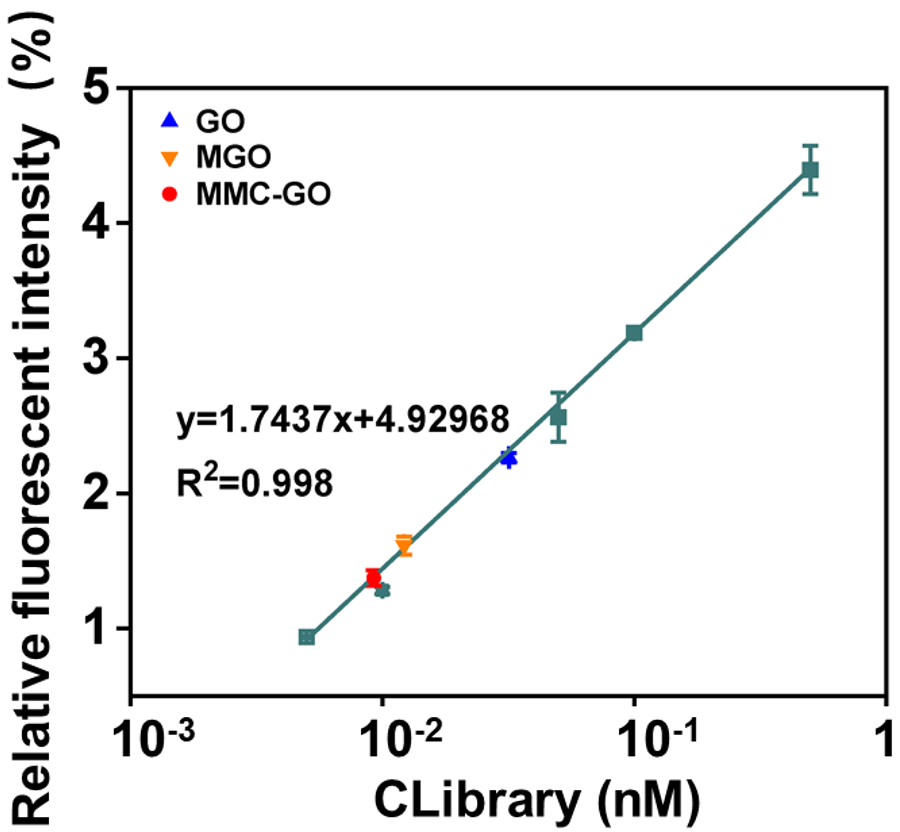


**Fig.S3.** The relationship between the concentrations of circular library (CLibrary) and their corresponding fluorescence intensity (marked in green). The SE of GO (blue dots), MGO (yellow dots) and MMC-GO (red dots) were calculated by the ratio of adsorbed circular ssDNA and unadsorbed circular ssDNA. The data represent the mean ± SD, n=3.


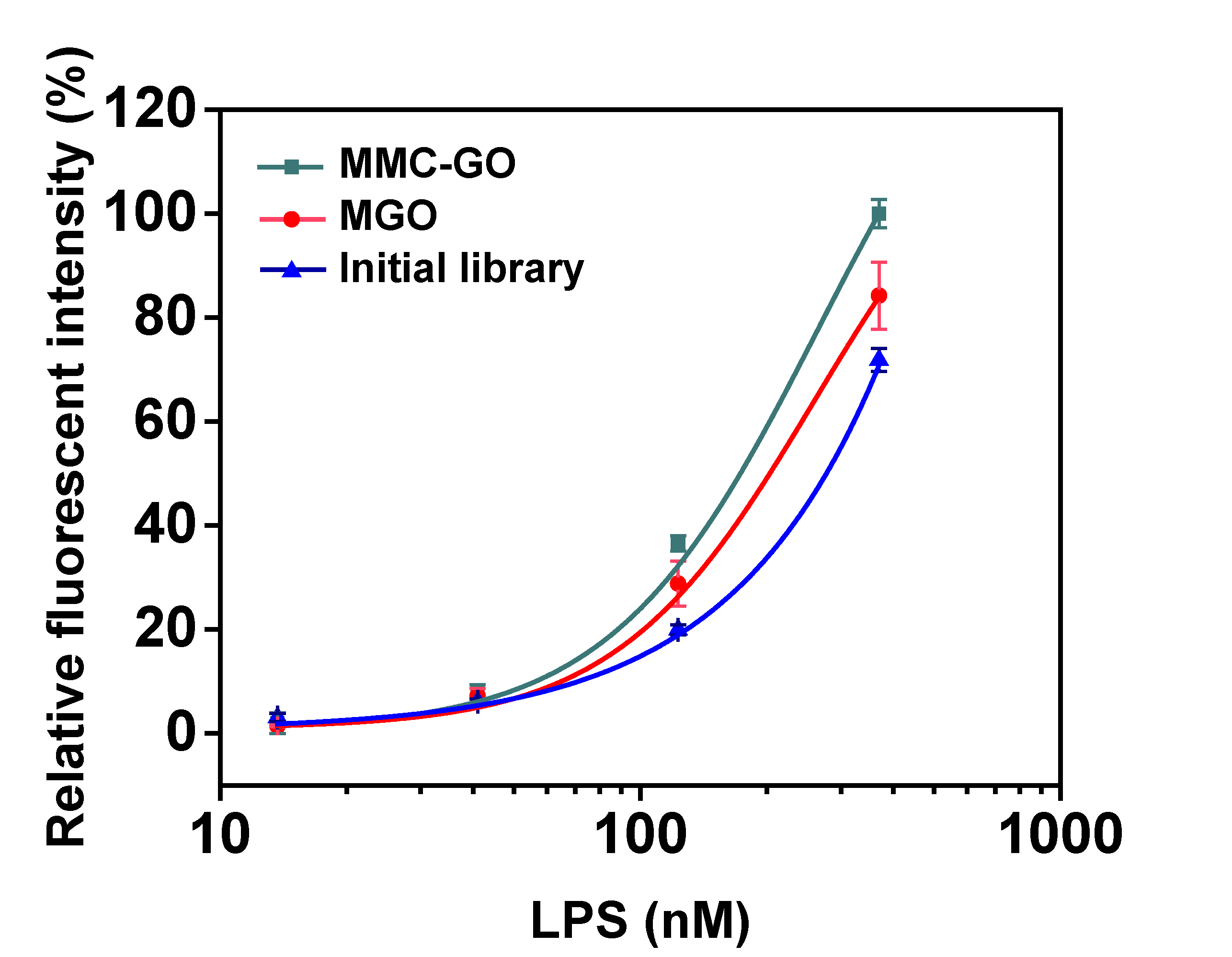


**Fig.S4.** Relative LPS binding capacity of the initial library (blue dots), the selected pools from MGO-based single-round of selection (red dots) and the selected pools from MMC-GO-based single-round of selection (green dots).


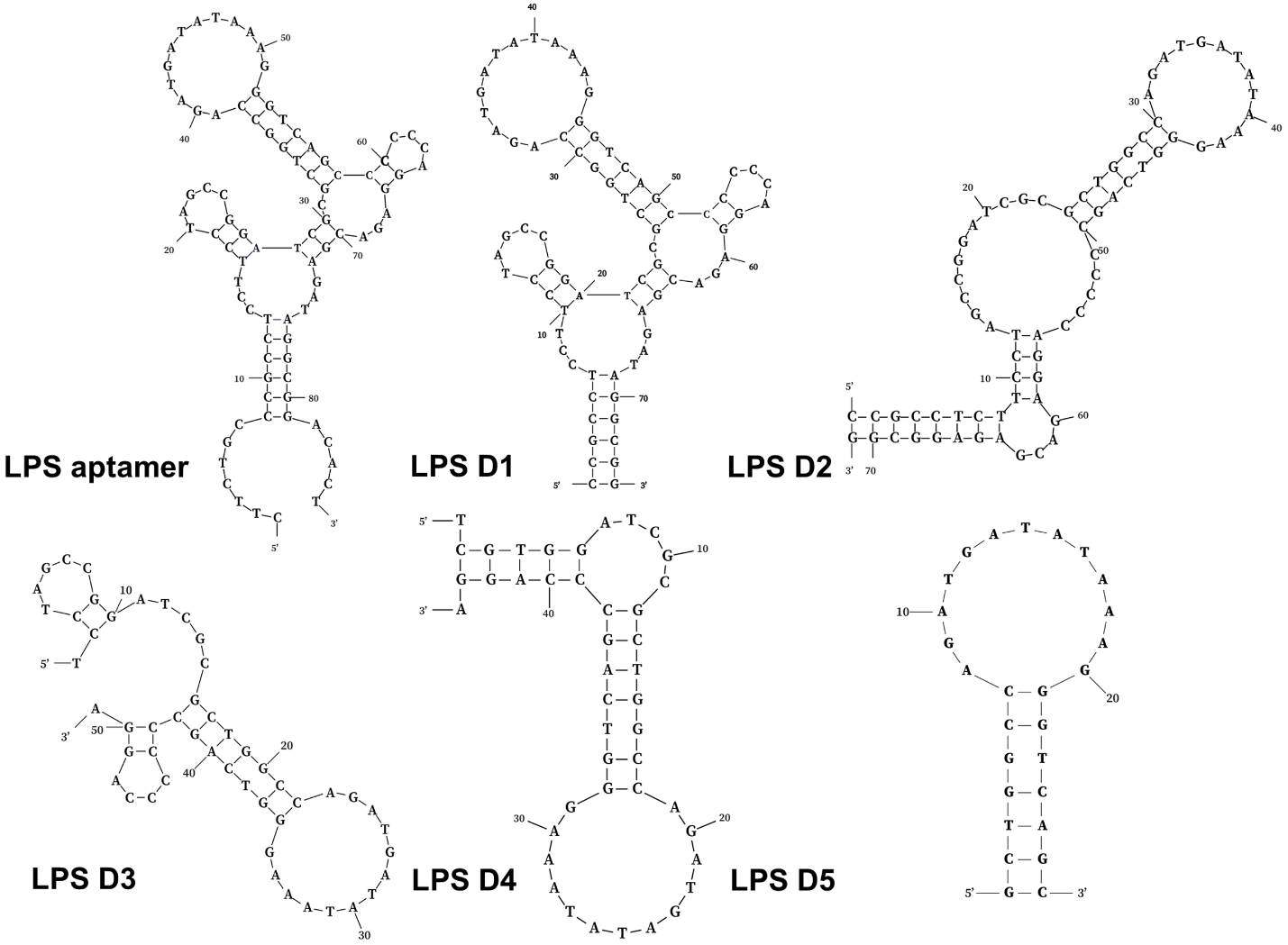


Fig.S5. The predicted secondary structure of the existing LPS aptamer and its truncation mutants.


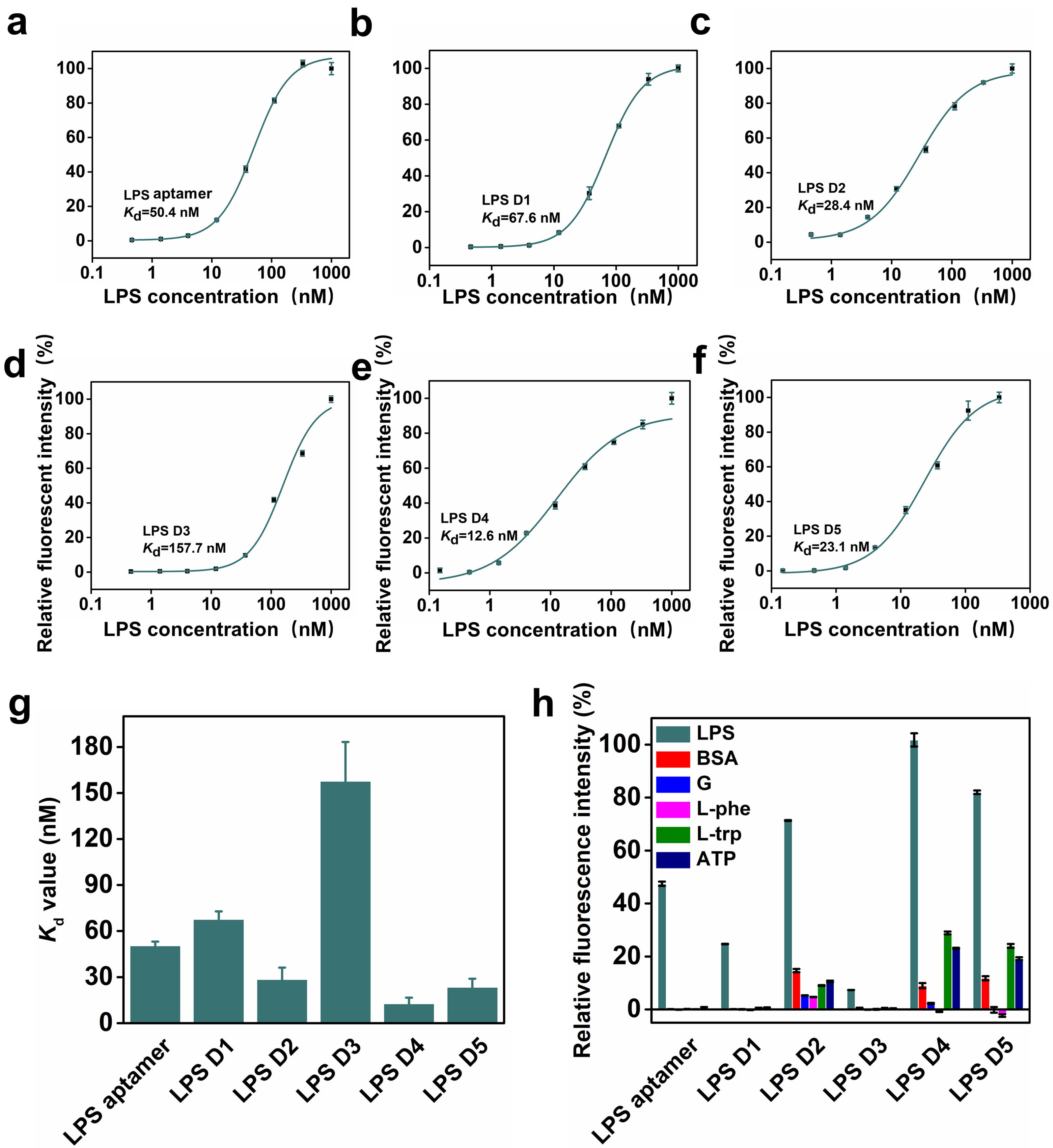


Fig.S6. Affinity and selectivity of the existing linear LPS aptamer and various truncated linear sequences. (a-f) The binding affinity of the existing LPS aptamer and various truncated linear aptamers toward LPS using MMC-GO-based binding assay (The data represent the mean ± SD, n=3). g) Comparison of *K*_d_ values of LPS aptamer and various truncated linear aptamers (The data represent the mean ± SD, n=3). h) The specificity of the LPS aptamer and various truncated linear aptamers toward LPS (The data represent the mean ± SD, n=3).


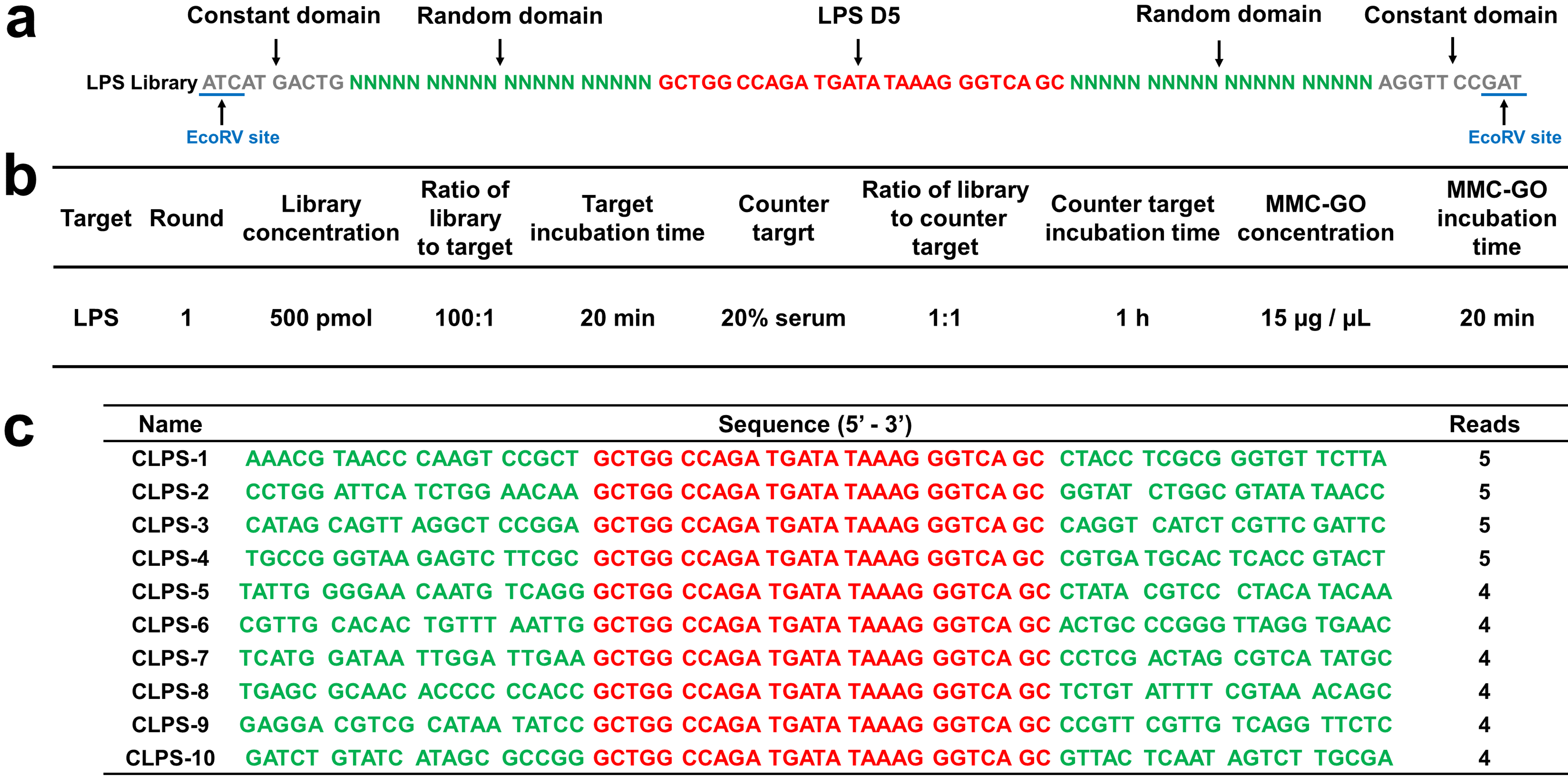


Fig.S7. Design of single-round of selection against LPS. a) The sequence of the LPS DNA library. b) LPS circular aptamer single-round of selection conditions. c) The sequence of top 10 LPS aptamer candidates.





**Fig.S8.** Relative binding activity of the top four circular aptamer sequences for LPS using MMC-GO-based binding assay (The data represent the mean ± SD, n=3).


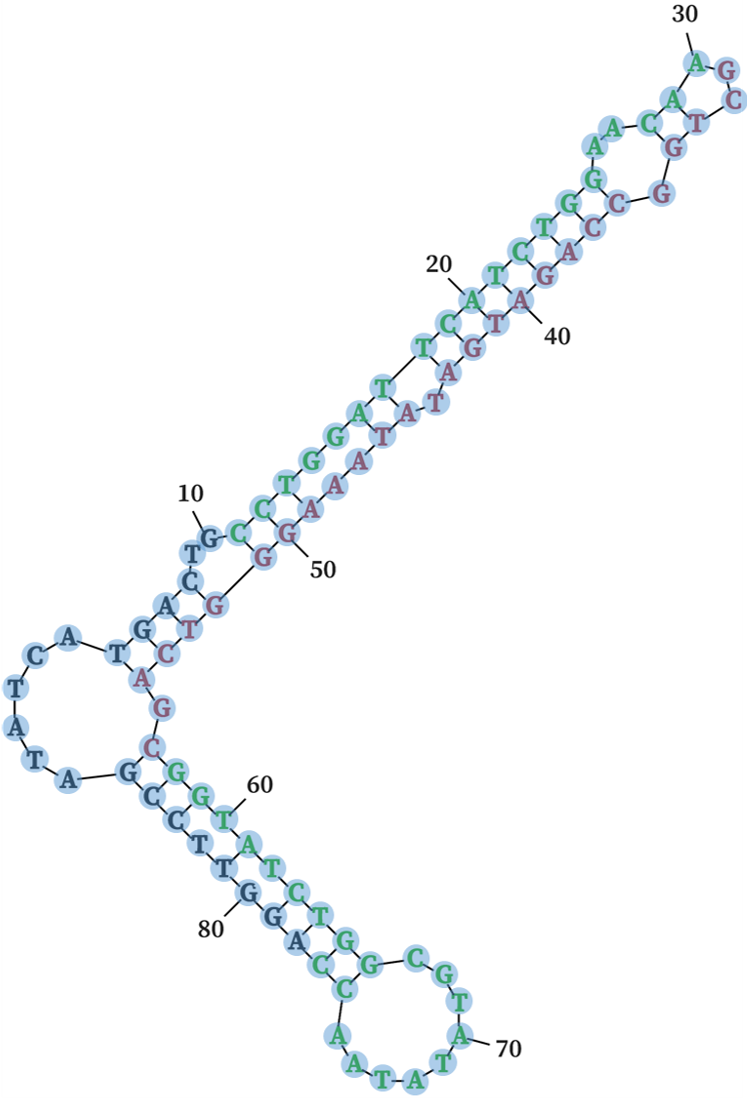


**Fig.S9.** Secondary structure of CLPS-2.


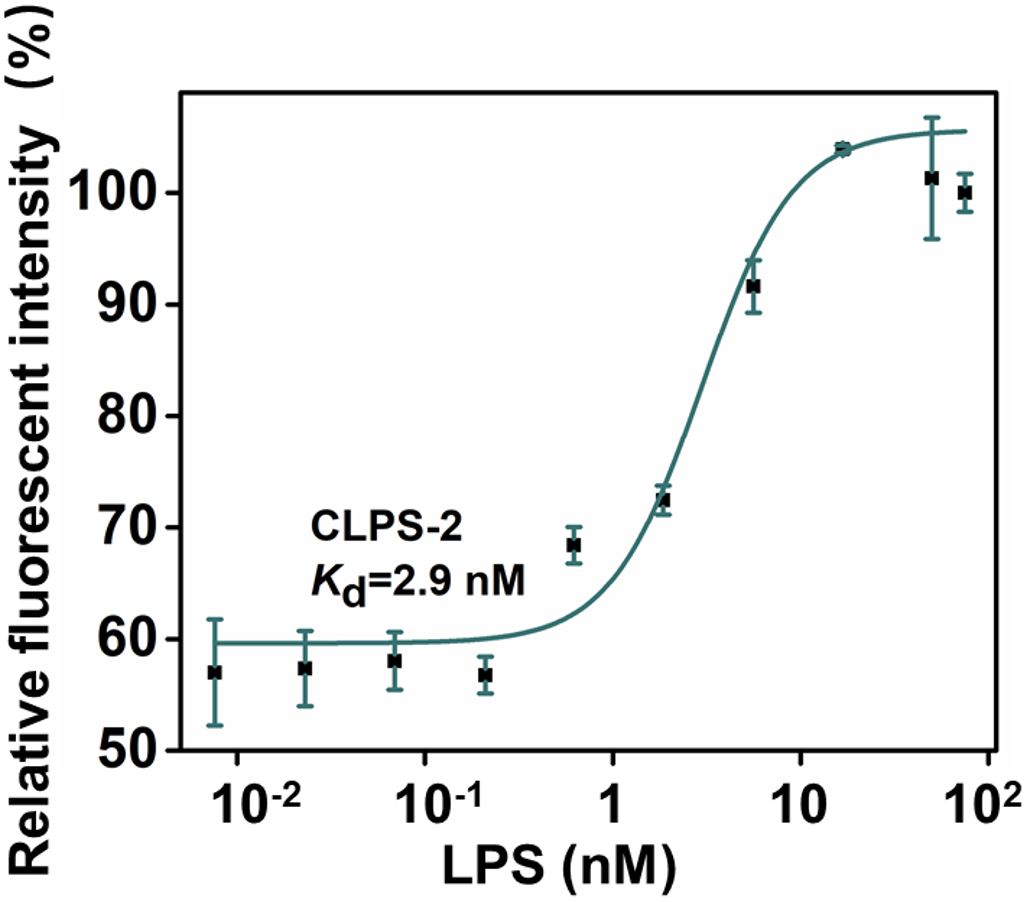


Fig.S10. Binding curve of CLPS-2 against LPS using MMC-GO-based binding assay (The data represent the mean ± SD, n=3).


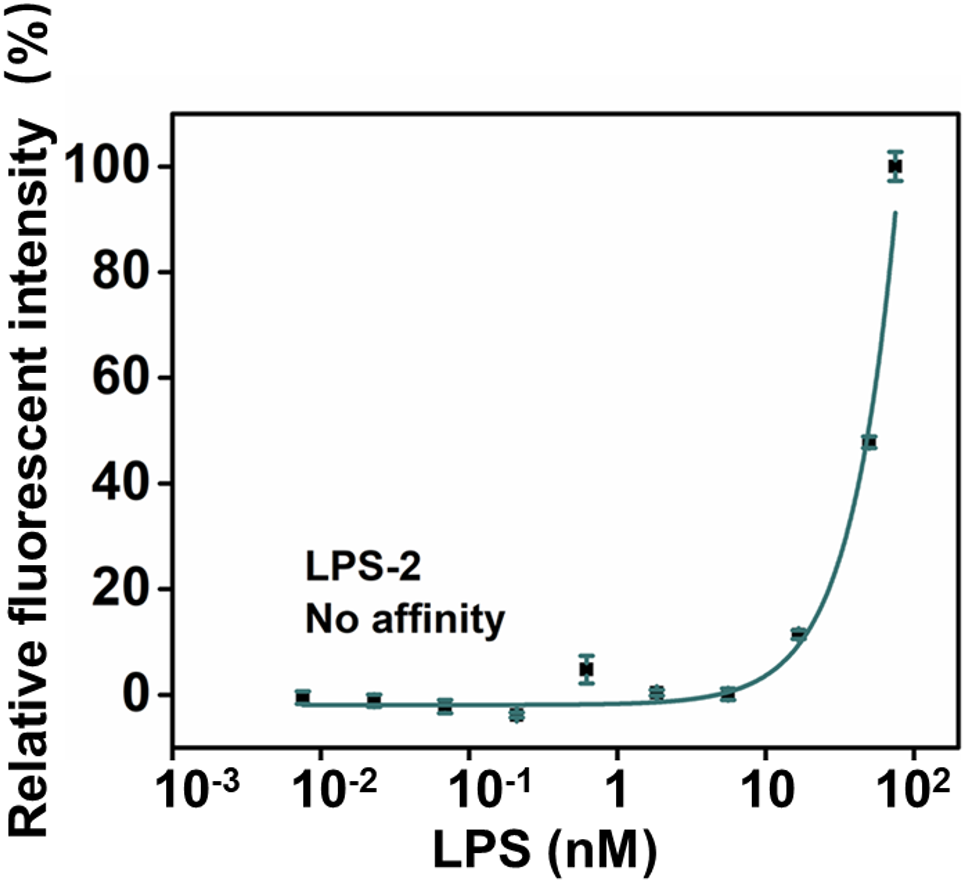


Fig.S11. Binding curve of LPS-2 (linear counterpart of CLPS-2) against LPS using MMC-GO-based fluorescent assay (The data represent the mean ± SD, n=3).


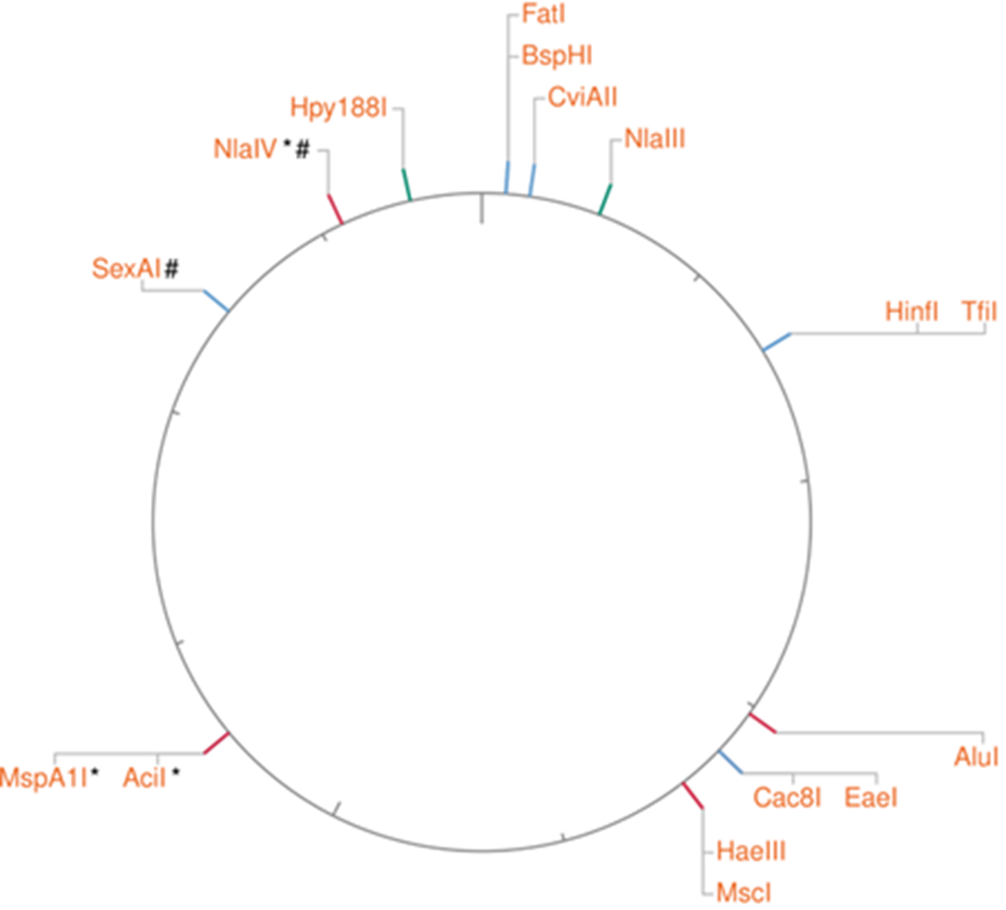


**Fig.S12.** Potential endonuclease cutting sites in CLPS-2 analyzed by NEBcutter (https://nc3.neb.com/NEBcutter/).


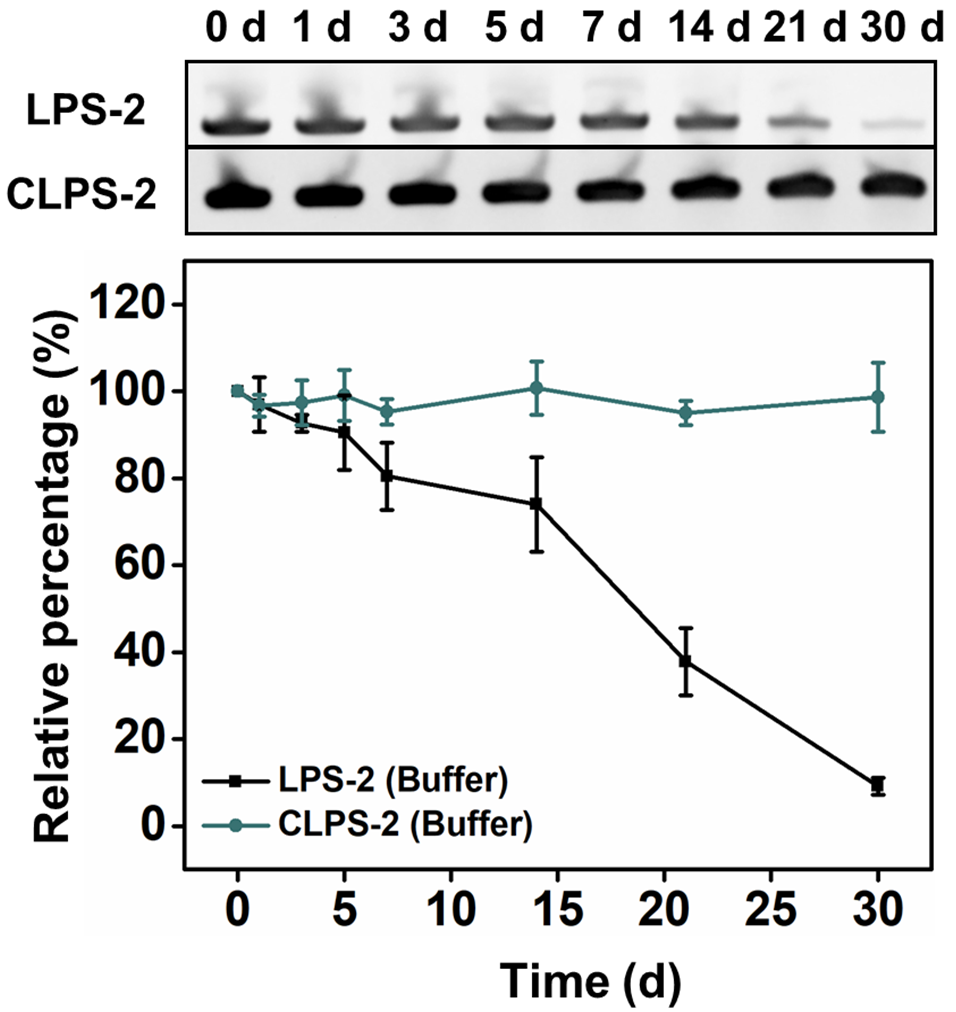


**Fig.S13.** Stability of CLPS-2 and LPS-2 in buffer at RT. Top: gel images of CLPS-2 and LPS-2 after RT exposure for the indicated period of time. The fraction of intact CLPS-2 and LPS-2 (relative to the zero time point) was plotted as a function of time.


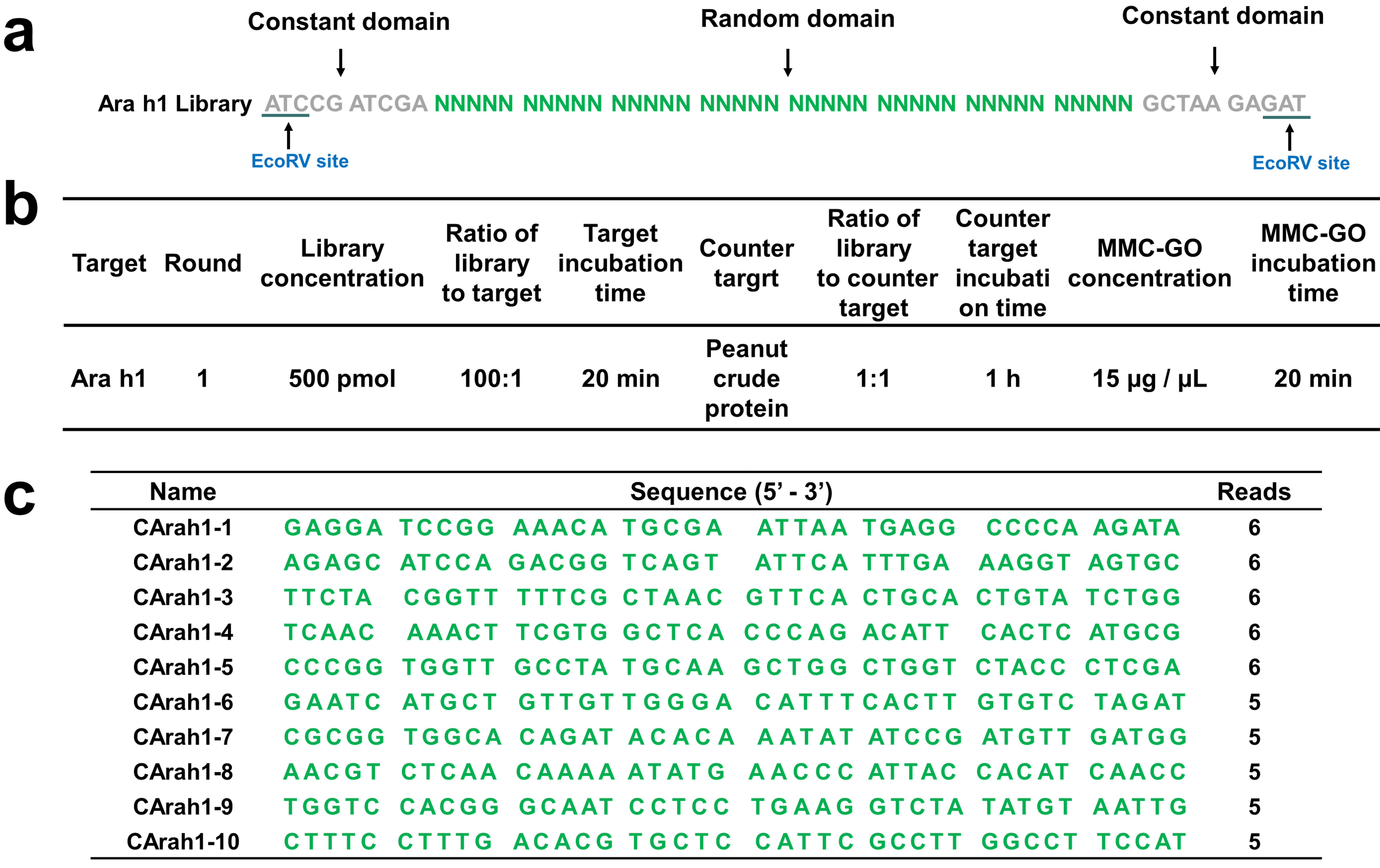


Fig.S14. Design of single-round of selection against Ara h1. a) The sequence of the Ara h1 DNA library. b) Ara h1 circular aptamer single-round of selection conditions. c) The sequence of top 10 Ara h1 aptamer candidates.


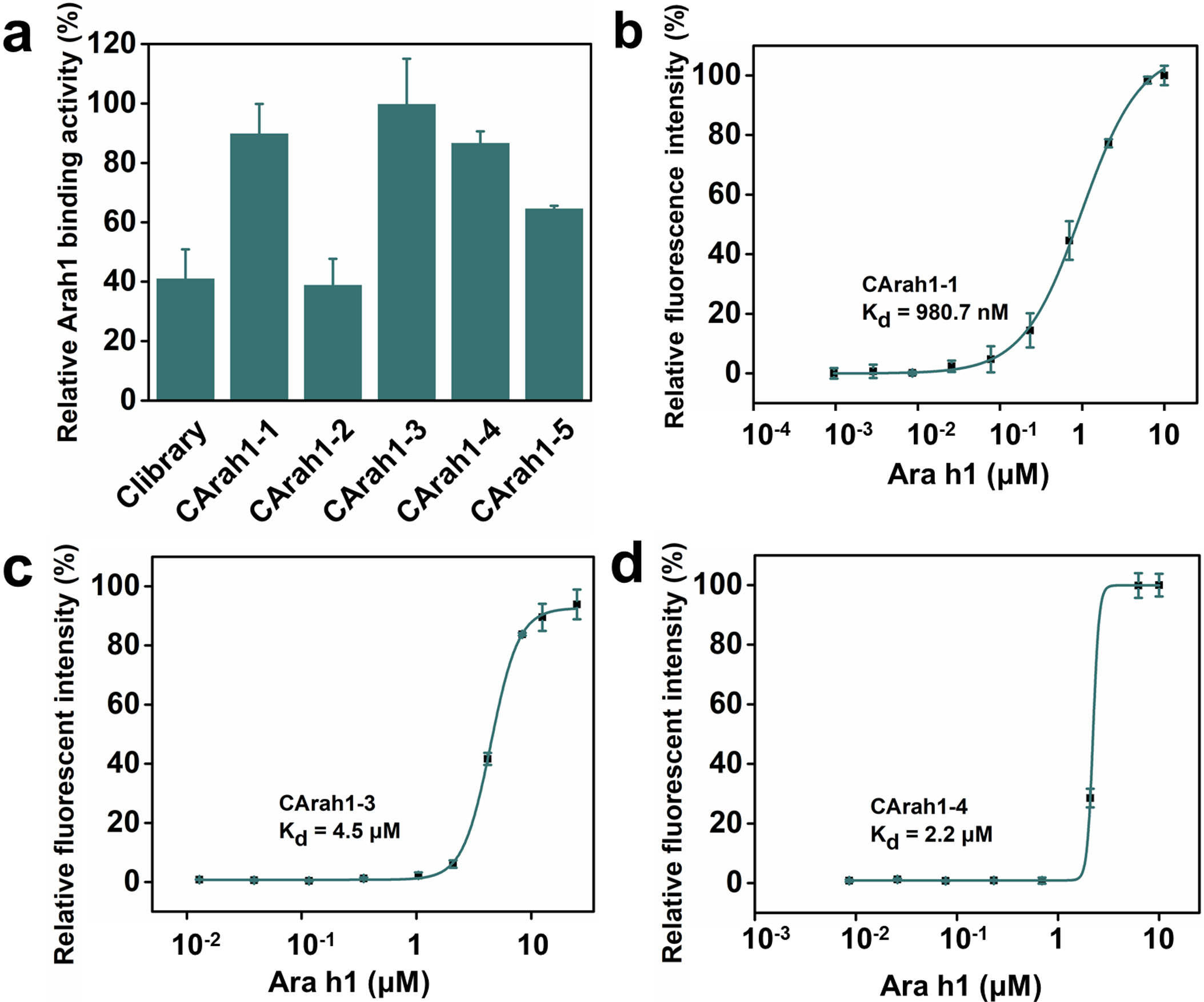


Fig.S15. Affinity analysis of the selected Ara h1 circular aptamers. a) Relative binding activity of the top five circular aptamer sequences for Ara h1 using MMC-GO-based binding assay (The data represent the mean ± SD, n=2). b-d) Binding curves of CArah1-1, CArah1-3 and CArah1-4 against Ara h1 using MMC-GO-based binding assay (The data represent the mean ± SD, n=2).


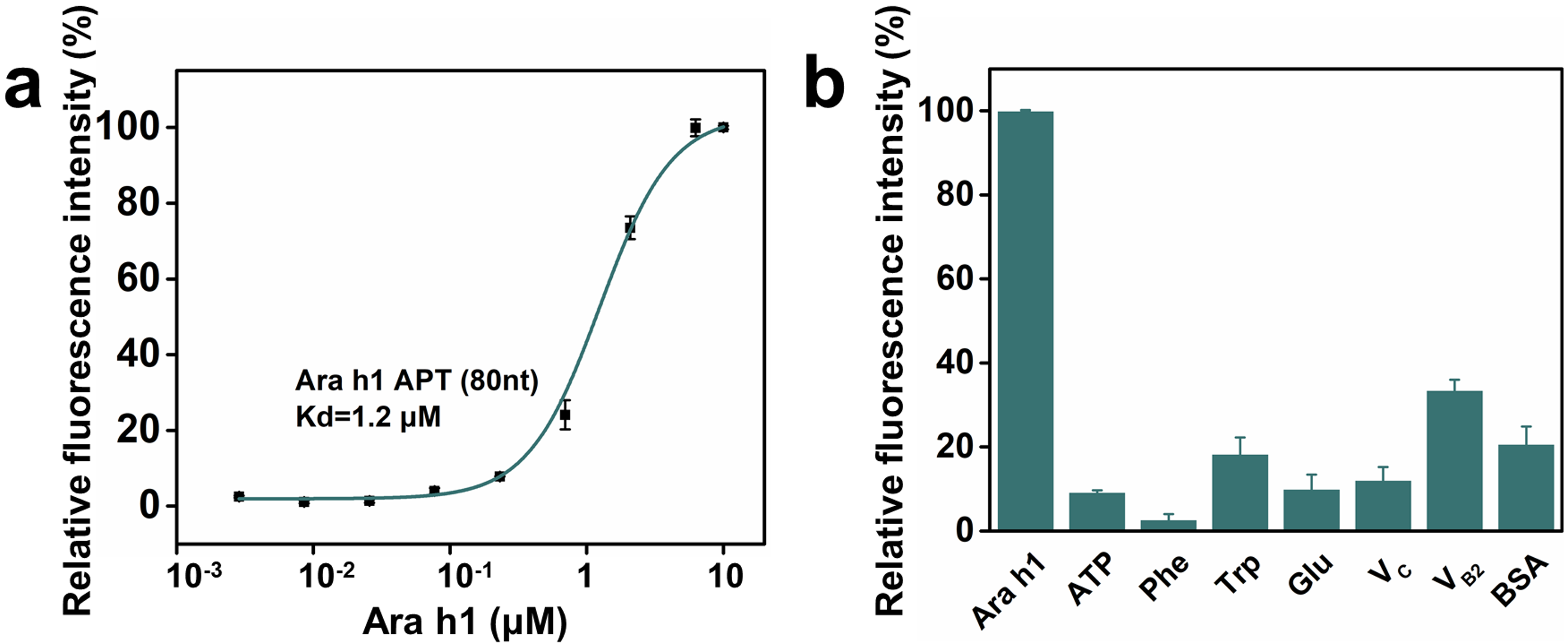


**Fig.S16.** a) Binding curve of the reported linear aptamer (80nt) against Ara h1 using MMC-GO-based binding assay (The data represent the mean ± SD, n=2). b) Characteristic of the specificity of Ara h1 APT (80nt) (The data represent the mean ± SD, n=2).


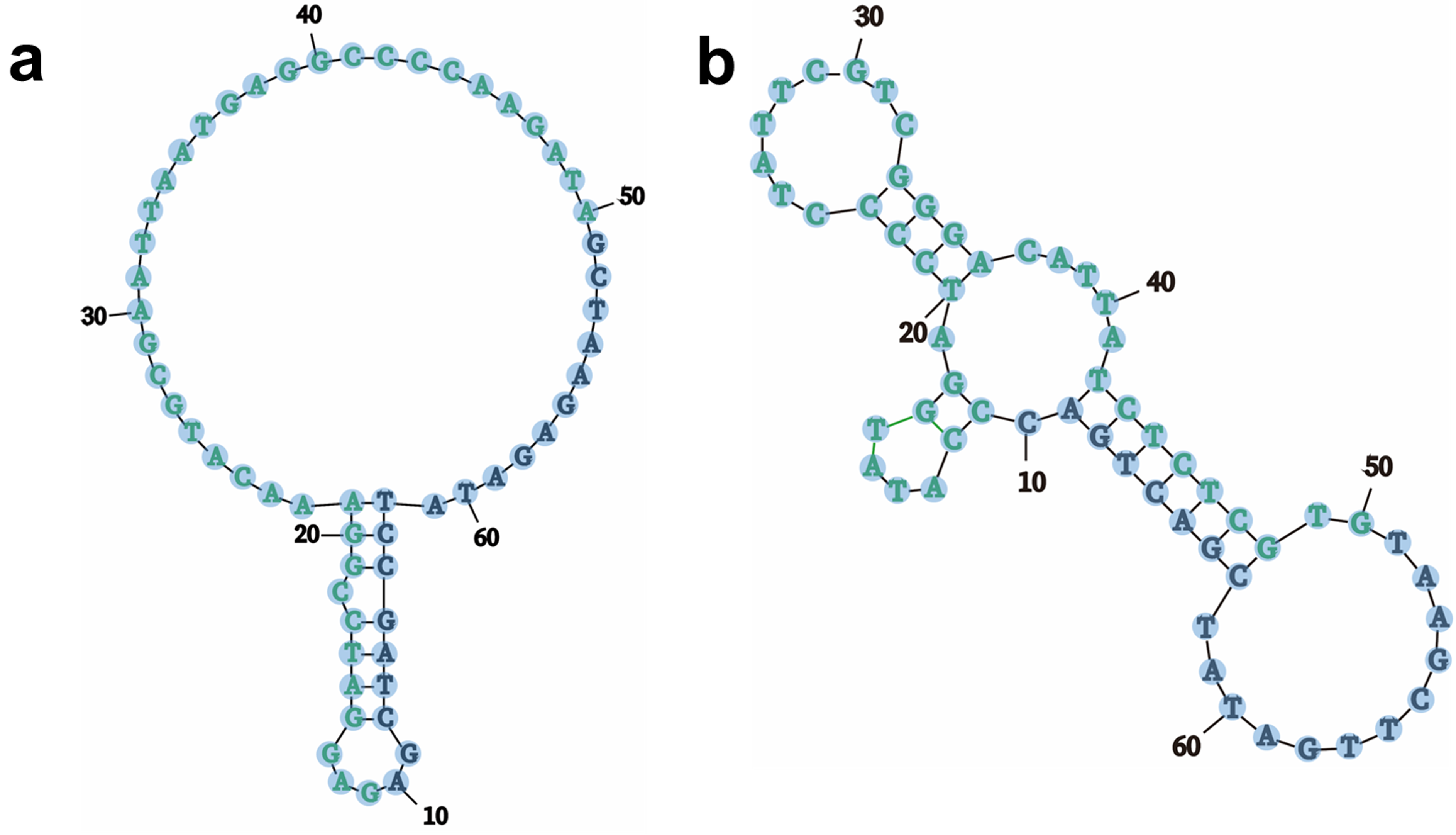


**Fig.S17.** Secondary structure of a) CArah1-1 and b) CAFB_1_-1.


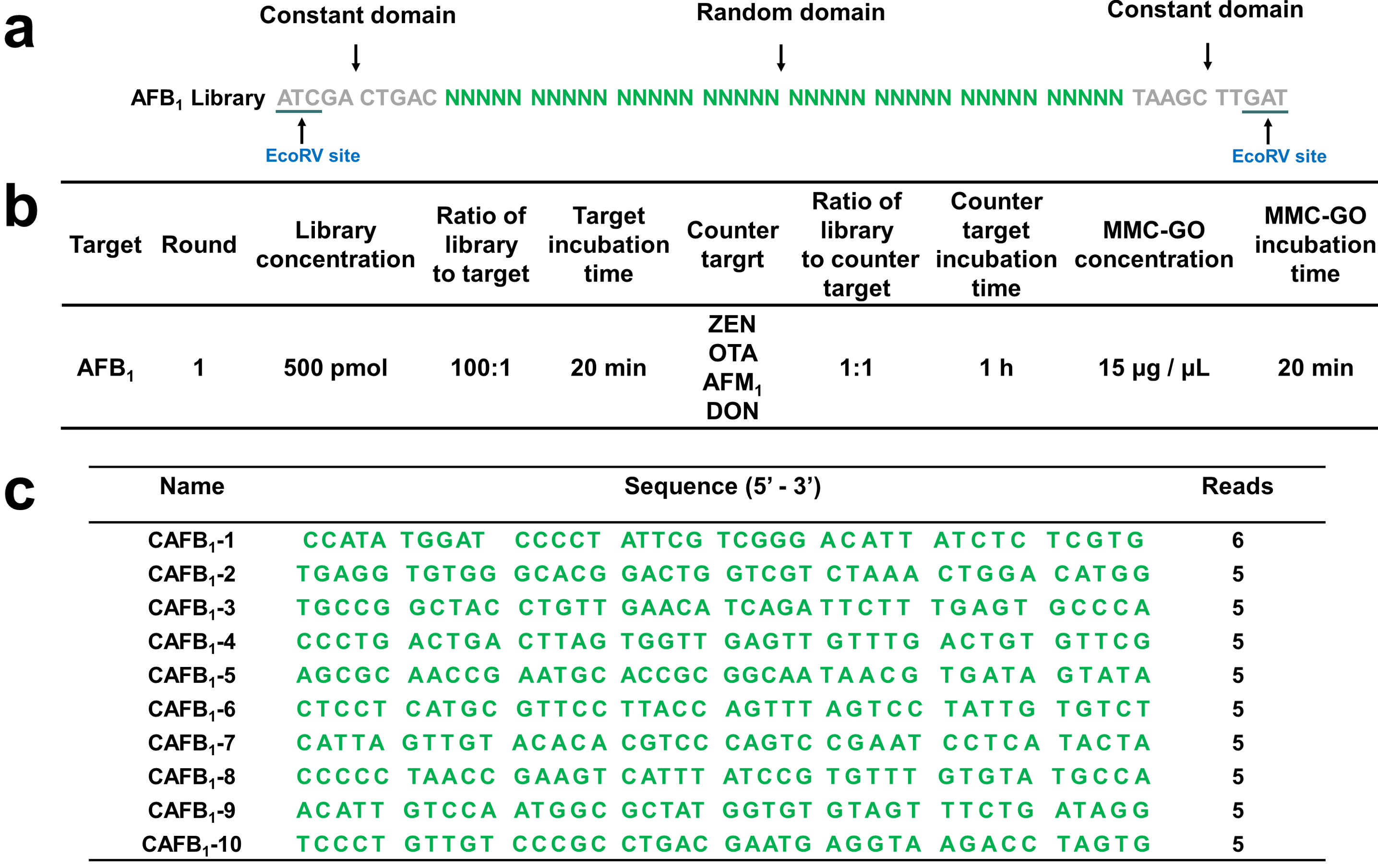


Fig.S18. Design of single-round of selection against AFB_1_. a) The sequence of the AFB_1_ DNA library. b) AFB_1_ circular aptamer single-round of selection conditions. c) The sequence of top 10 AFB_1_ aptamer candidate.





Fig.S19. Relative binding activity of the top one circular aptamer sequence for AFB_1_ using MMC-GO-based binding assay (The data represent the mean ± SD, n=3).


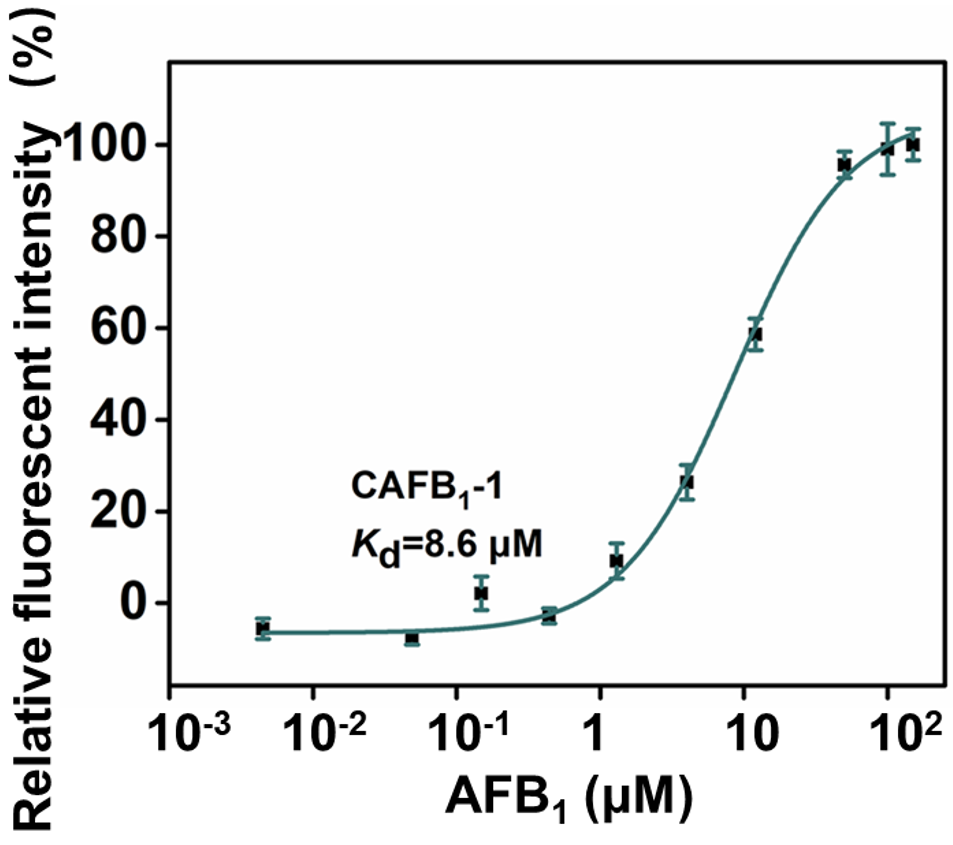


**Fig.S20.** Binding curve of CAFB_1_-1 against AFB_1_ using MMC-GO-based binding assay (The data represent the mean ± SD, n=3).


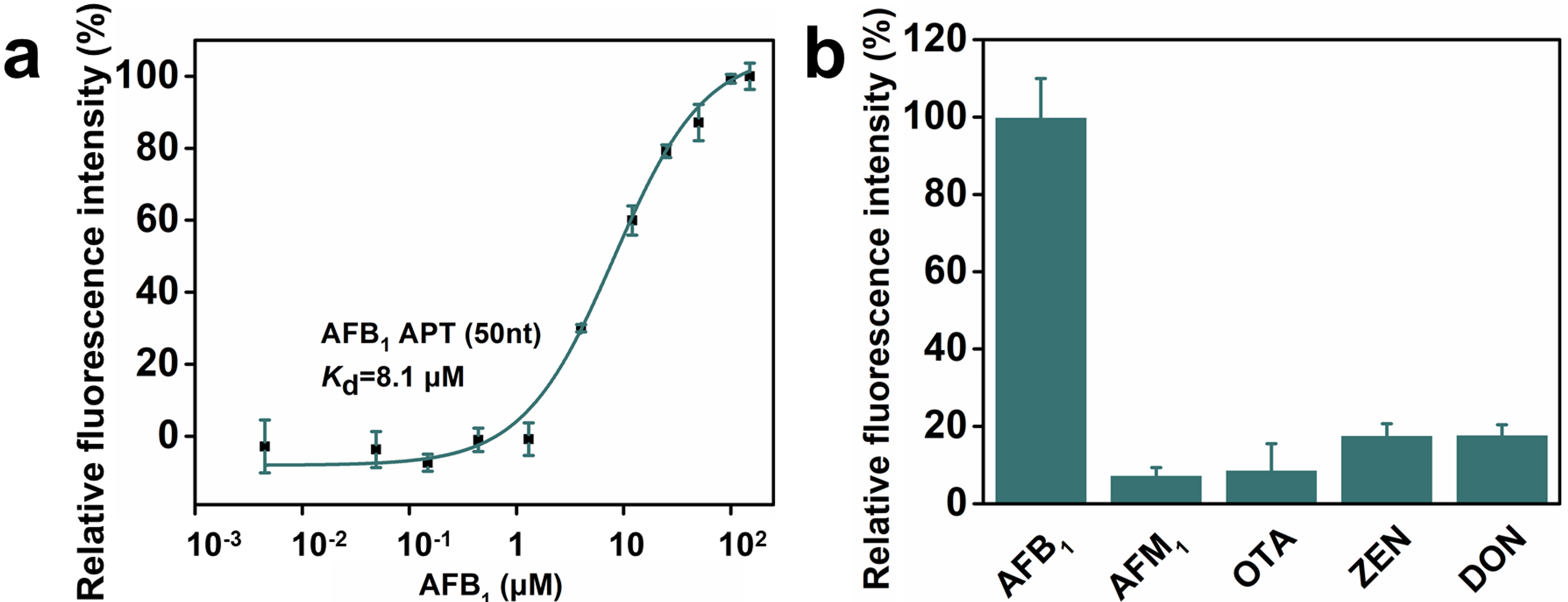


**Fig.S21.** a) Binding curve of the reported linear aptamer (50nt) against AFB_1_ using MMC-GO-based fluorescent assay (The data represent the mean ± SD, n=3). b) Characteristic of the specificity of AFB_1_ APT (50nt) (The data represent the mean ± SD, n=3).

| Name of DNA oligonucleotide | Sequence (5’ - 3’) |
| --- | --- |
| LPS Library | ATCAT GACTG NNNNN NNNNN NNNNN NNNNN GCTGG CCAGA TGATA TAAAG GGTCA GCNNN NNNNN NNNNN NNNNN NNAGG TTCCG AT |
| LPS LT1 | CAGTC ATGAT ATCGG AACCT |
| LPS LT2 | AGGTT CCGAT ATCAT GACTG |
| LPS F1 | TACTG CCGGA ATCAT GACTG |
| LPS F2 | CATGC ATTGG ATCGG AACCT |
| LPS library-random | ATC GT ACTCG NNNNN NNNNN NNNNN NNNNN NNNNN NNNNN NNNNN NNNNN AATCT CG GAT |
| LPS Lib-Random LT1 | CGAGT ACGAT ATCCG AGATT |
| LPS Lib-Random LT2 | AATCT CGGAT ATCGT ACTCG |
| LPS Lib-Random F1 | ACAGA AGTCT ATC GT ACTCG |
| LPS Lib-Random F2 | TACGA GTCAT ATCCG AGATT |
| Ara h1 Library | ATCCG ATCGA NNNNN NNNNN NNNNN NNNNN NNNNN NNNNN NNNNN NNNNN GCTAA GAGAT |
| Ara h1 LT1 | TCGAT CGGAT ATCTC TTAGC |
| Ara h1 LT2 | GCTAA GAGAT ATCCG ATCGA |
| Ara h1 F1 | CGTAT GTCCA ATCCG ATCGA |
| Ara h1 F2 | AACCT GTGCA ATCTC TTAGC |
| AFB1 Library | ATCGA CTGAC NNNNN NNNNN NNNNN NNNNN NNNNN NNNNN NNNNN NNNNN TAAGC TTGAT |
| AFB1 LT1 | GTCAG TCGAT ATCAA GCTTA |
| AFB1 LT2 | TAAGC TTGAT ATCGA CTGAC |
| AFB1 F1 | TTCCT ATACC ATC GA CTGAC |
| AFB1 F2 | CCGTT ATGCA ATCAA GCTTA |
| LPS | CTTCT GCCCG CCTCC TTCCT AGCCG GATCG CGCTG GCCAG ATGAT ATAAA GGGTC AGCCC CCCAG GAGAC GAGAT AGGCG GACAC T |
| LPS D1 | CCGCC TCCTT CCTAG CCGGA TCGCG CTGGC CAGAT GATAT AAAGG GTCAG CCCCC CAGGA GACGA GATAG GCGG |
| LPS D2 | CCGCC TCTTC CTAGC CGGAT CGCGC TGGCC AGATG ATATA AAGGG TCAGC CCCCC AGGAG ACGAG AGGCG G |
| LPS D3 | TCCTA GCCGG ATCGC GCTGG CCAGA TGATA TAAAG GGTCA GCCCC CCAGG A |
| LPS D4 | TCCTG GATCG CGCTG GCCAG ATGAT ATAAA GGGTC AGCCC AGGA |
| LPS D5 | GCTGG CCAGA TGATA TAAAG GGTCA GC |
| CLPS-1 | ATCAT GACTG AAACG TAACC CAAGT CCGCT GCTGG CCAGA TGATA TAAAG GGTCA GCCTA CCTCG CGGGT GTTCT TAAGG TTCCG AT |
| CLPS-2 | ATCAT GACTG CCTGG ATTCA TCTGG AACAA GCTGG CCAGA TGATA TAAAG GGTCA GCGGT ATCTG GCGTA TATAA CCAGG TTCCG AT |
| CLPS-3 | ATCAT GACTG CATAG CAGTT AGGCT CCGGA GCTGG CCAGA TGATA TAAAG GGTCA GCCAG GTCAT CTCGT TCGAT TCAGG TTCCG AT |
| CLPS-4 | ATCAT GACTG TGCCG GGTAA GAGTC TTCGC GCTGG CCAGA TGATA TAAAG GGTCA GCCGT GATGC ACTCA CCGTA CTAGG TTCCG AT |
| CArah1-1 | ATCCG ATCGA GAGGA TCCGG AAACA TGCGA ATTAA TGAGG CCCCA AGATA GCTAA GAGAT |
| CArah1-2 | ATCCG ATCGA AGAGC ATCCA GACGG TCAGT ATTCA TTTGA AAGGT AGTGC GCTAA GAGAT |
| CArah1-3 | ATCCG ATCGA TTCTA CGGTT TTTCG CTAAC GTTCA CTGCA CTGTA TCTGG GCTAA GAGAT |
| CArah1-4 | ATCCG ATCGA TCAAC AAACT TCGTG GCTCA CCCAG ACATT CACTC ATGCG GCTAA GAGAT |
| CArah1-5 | ATCCG ATCGA CCCGG TGGTT GCCTA TGCAA GCTGG CTGGT CTACC CTCGA GCTAA GAGAT |
| CAFB_1_-1 | ATCGA CTGAC CCATA TGGAT CCCCT ATTCG TCGGG ACATT ATCTC TCGTG TAAGC TTGAT |
| AFB_1_ APT (50 nt) | GTTGG GCACG TGTTG TCTCT CTGTG TCTCG TGCCC TTCGC TAGGC CCACA |
| Ara h1 APT (80nt) | TCGCA CATTC CGCTT CTACC GGGGG GGTCG AGCTG AGTGG ATGCG AATCT GTGGG TGGGC CGTAA GTCCG TGTGT GCGAA |

Table S1. Sequences of DNA oligonucleotides used in this work.

| Oligonucleotide | T_m_ (℃) |
| --- | --- |
| CLPS-2 | 66 ± 0.9 |
| LPS-2 | 59 ± 0.4 |

Table S2. Melting temperatures of CLPS-2 and LPS-2.
