# Supplementary figures and images for: Single-Round Circular Aptamer Discovery Using Bioinspired Magnetosome-Like Magnetic Chain Cross-Linked Graphene Oxide
Source: Research (Wash D C). 2024 May 1;7:0372. doi: 10.34133/research.0372 (PMC11062507; doi:10.34133/research.0372)

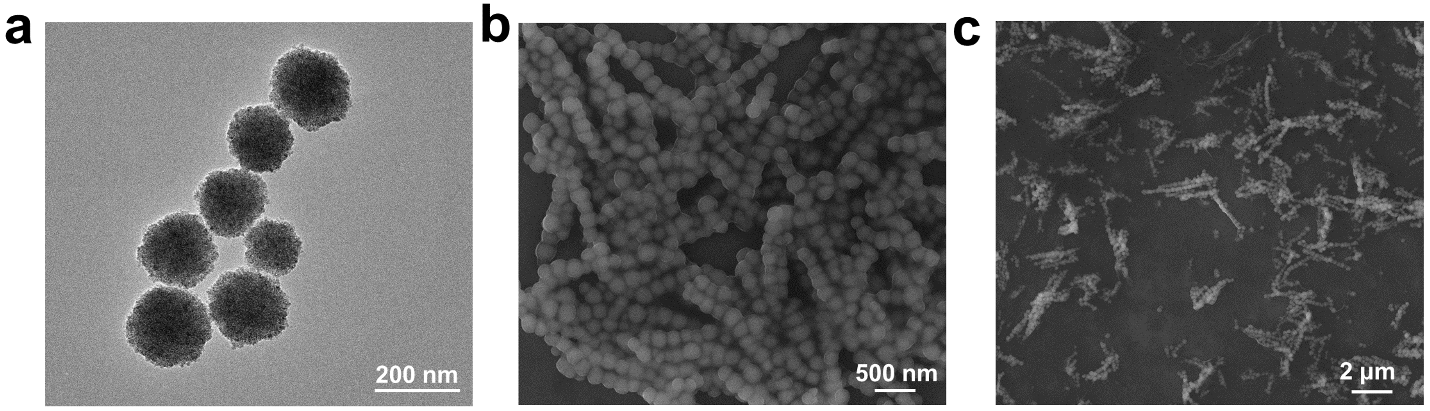

Supplement: Supplementary 1 — Supplementary Text Figs. S1 to S21 Table S1 and S2 [file research.0372.f1.zip › Figure S1.tif]

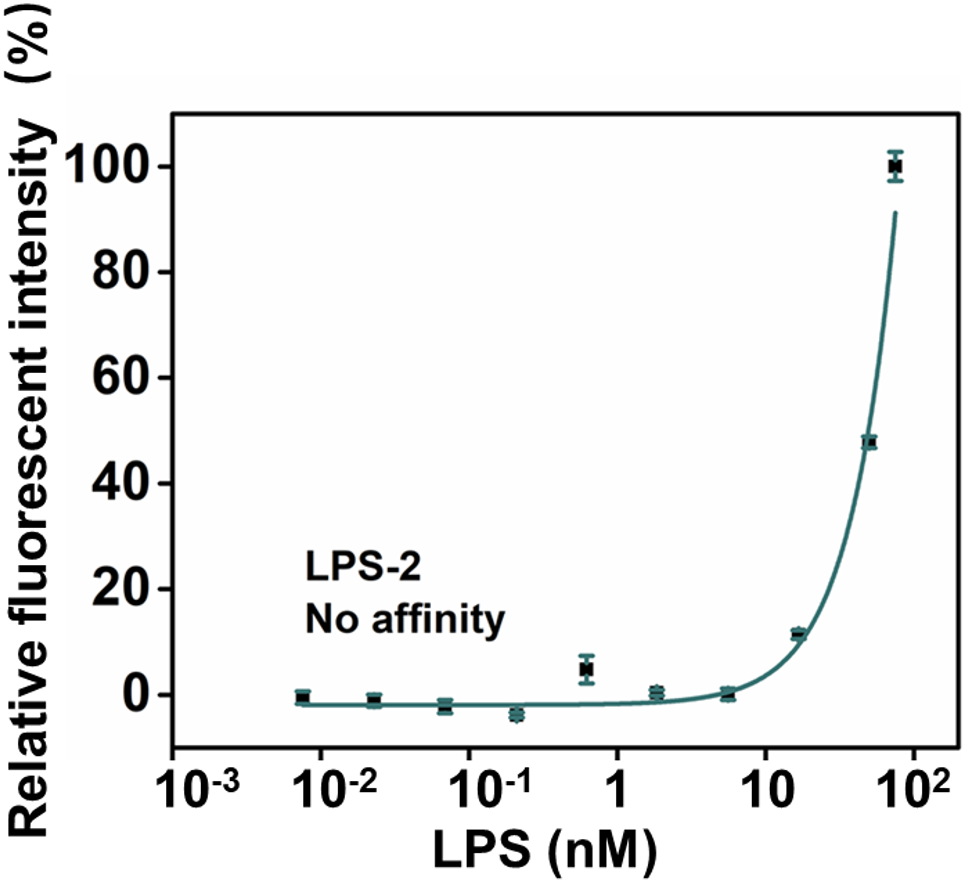

Supplement: Supplementary 1 — Supplementary Text Figs. S1 to S21 Table S1 and S2 [file research.0372.f1.zip › Figure S10.tif]

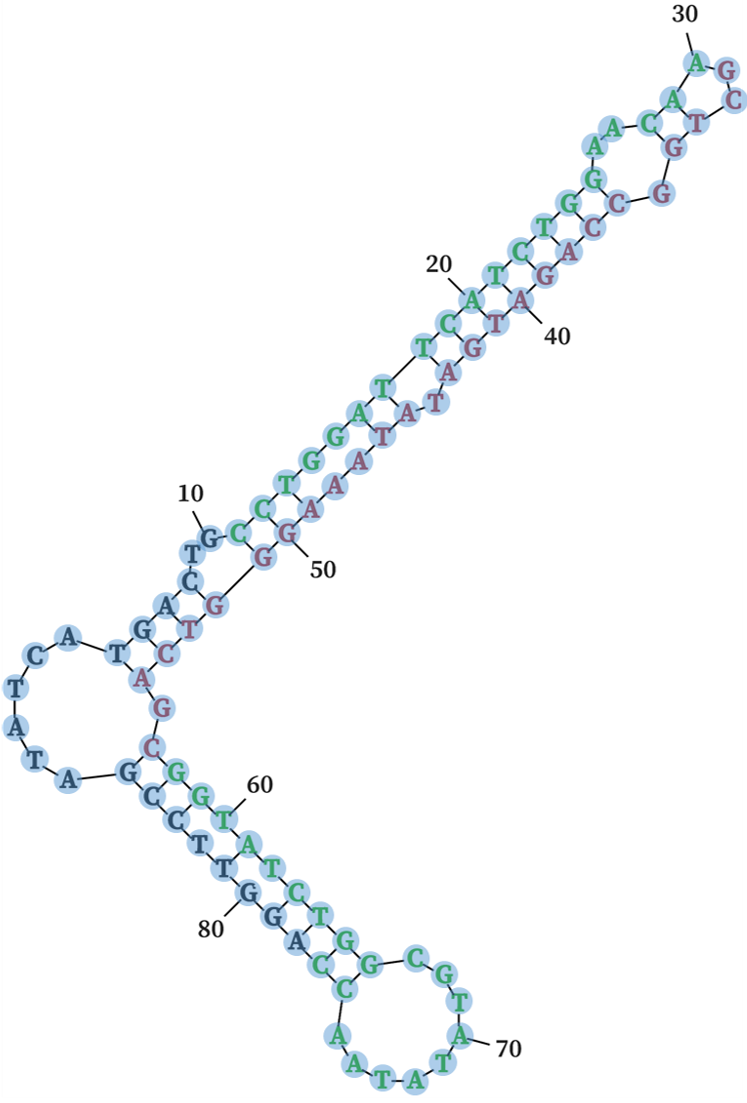

Supplement: Supplementary 1 — Supplementary Text Figs. S1 to S21 Table S1 and S2 [file research.0372.f1.zip › Figure S11.tif]

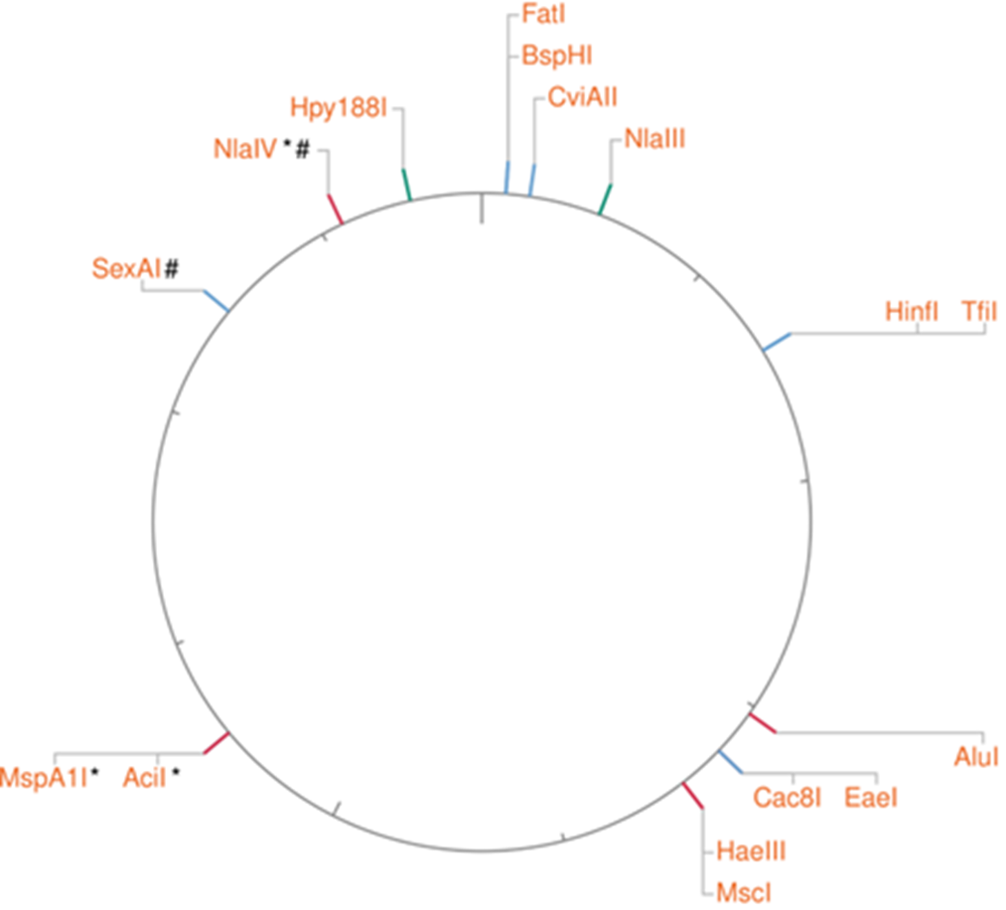

Supplement: Supplementary 1 — Supplementary Text Figs. S1 to S21 Table S1 and S2 [file research.0372.f1.zip › Figure S12.tif]

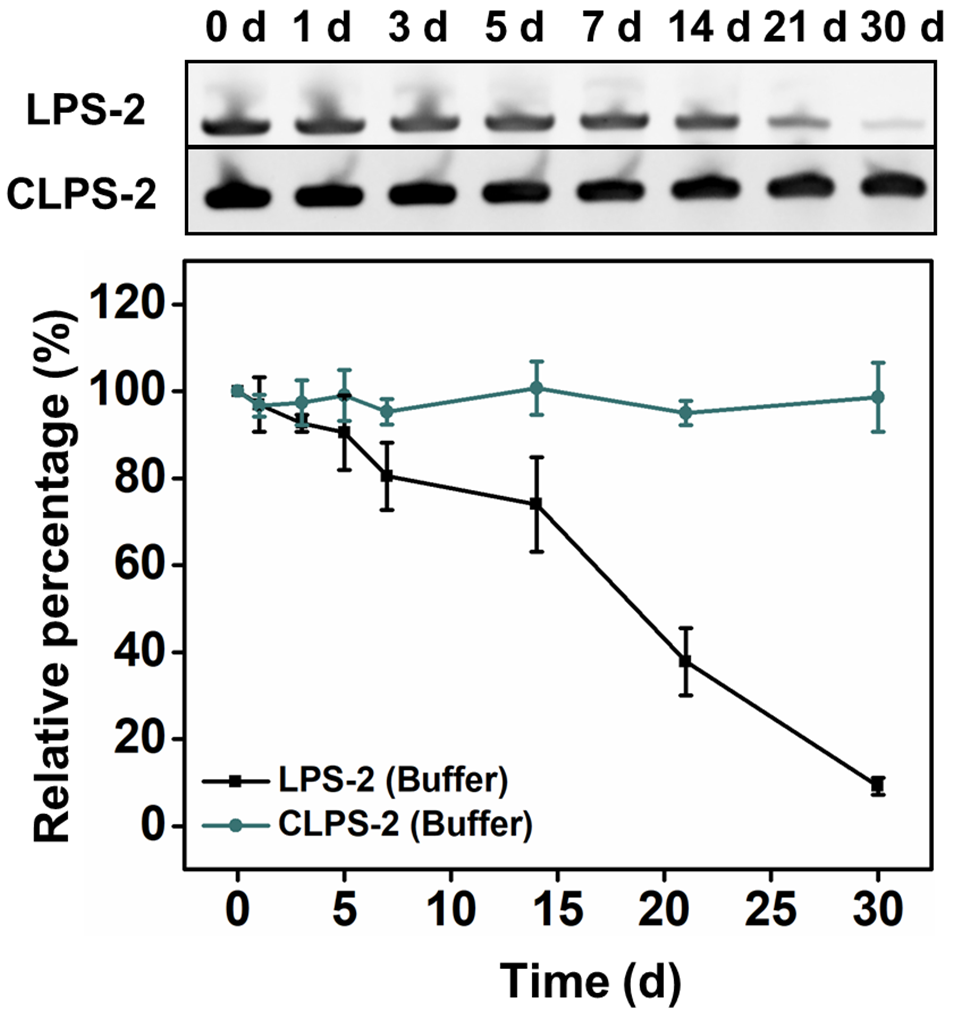

Supplement: Supplementary 1 — Supplementary Text Figs. S1 to S21 Table S1 and S2 [file research.0372.f1.zip › Figure S13.tif]

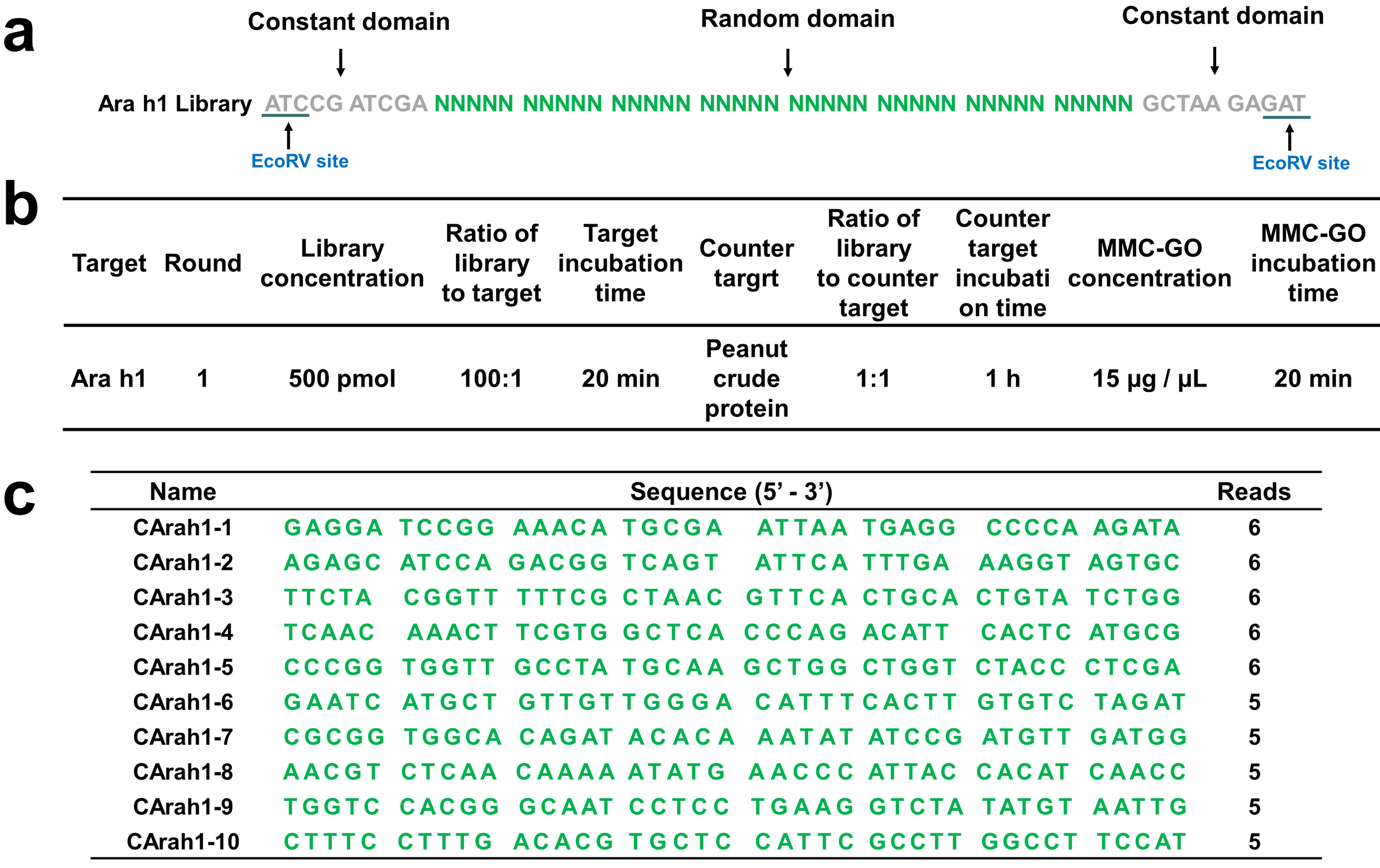

Supplement: Supplementary 1 — Supplementary Text Figs. S1 to S21 Table S1 and S2 [file research.0372.f1.zip › Figure S14.tif]

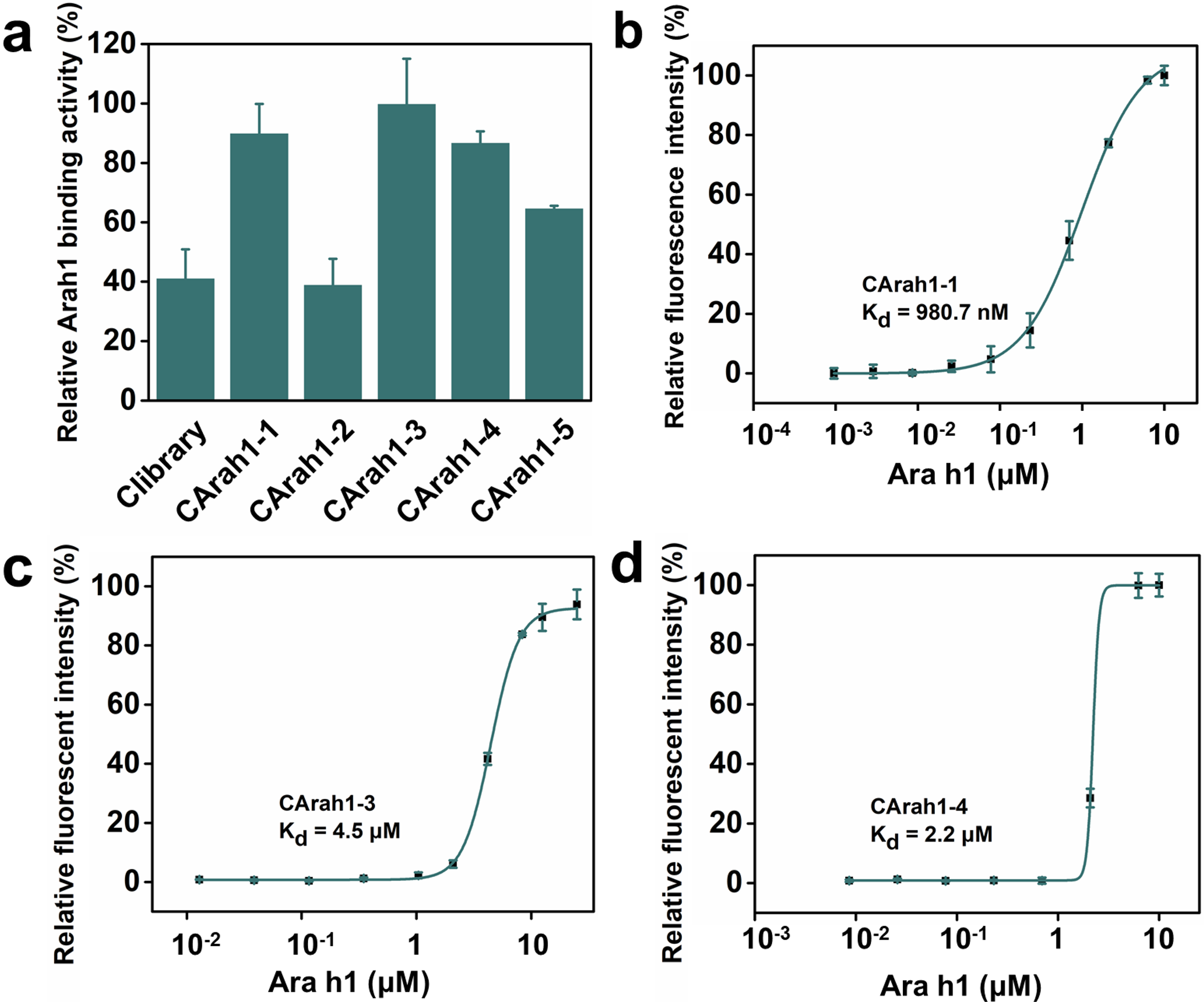

Supplement: Supplementary 1 — Supplementary Text Figs. S1 to S21 Table S1 and S2 [file research.0372.f1.zip › Figure S15.tif]

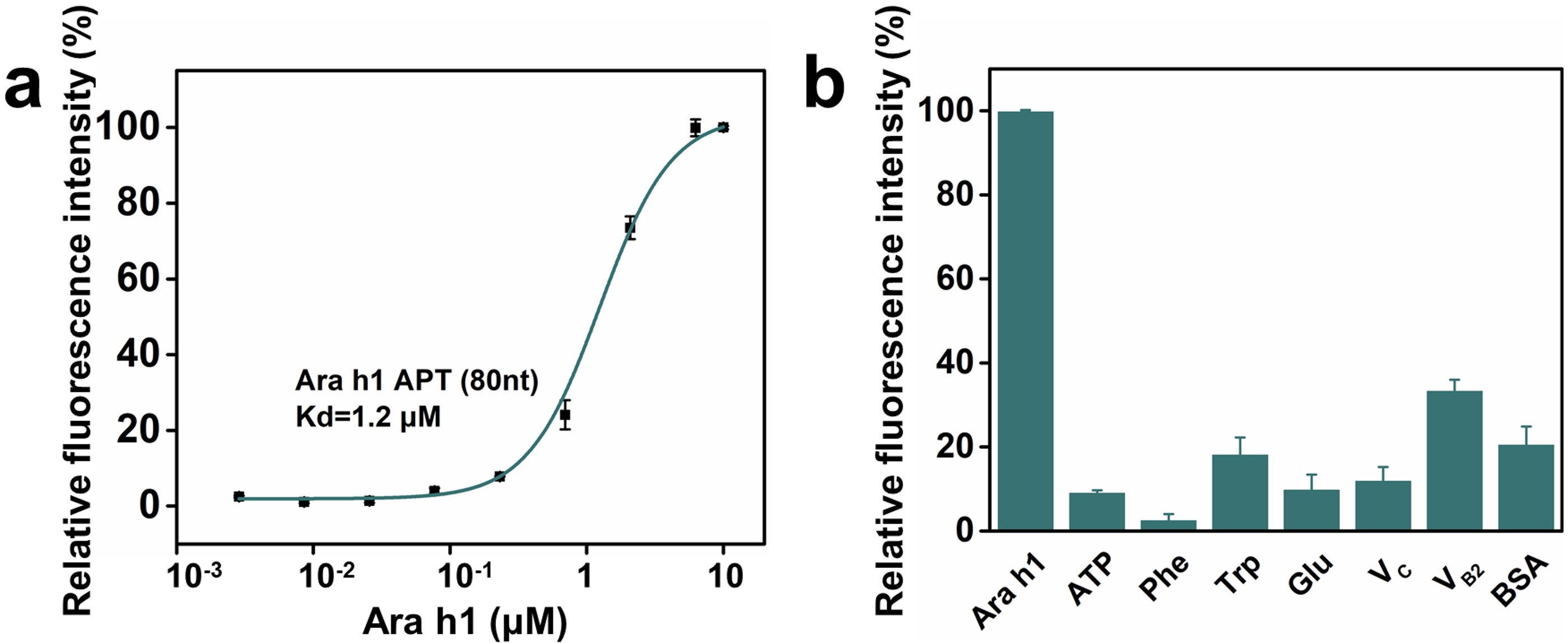

Supplement: Supplementary 1 — Supplementary Text Figs. S1 to S21 Table S1 and S2 [file research.0372.f1.zip › Figure S16.tif]

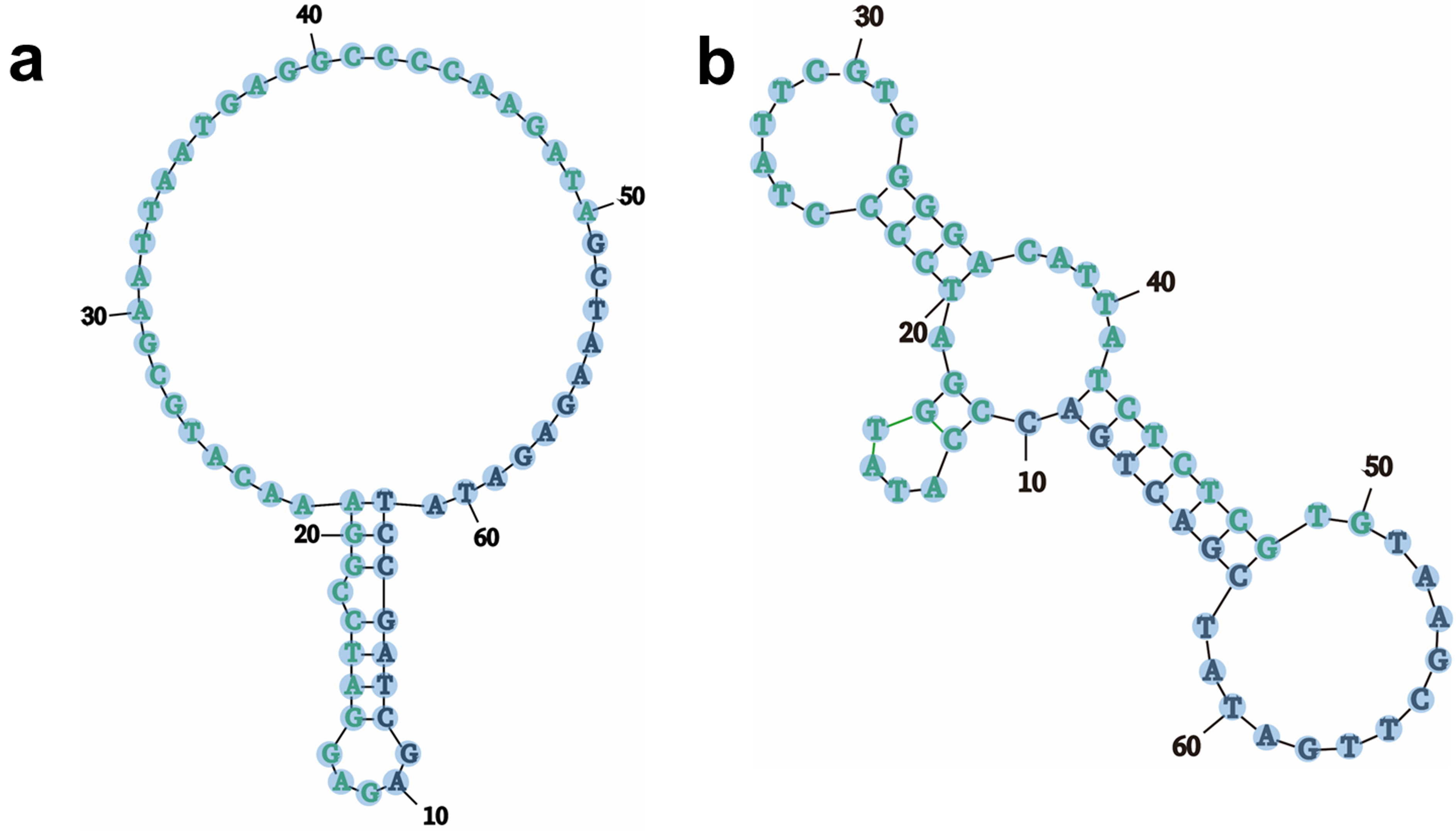

Supplement: Supplementary 1 — Supplementary Text Figs. S1 to S21 Table S1 and S2 [file research.0372.f1.zip › Figure S17.tif]

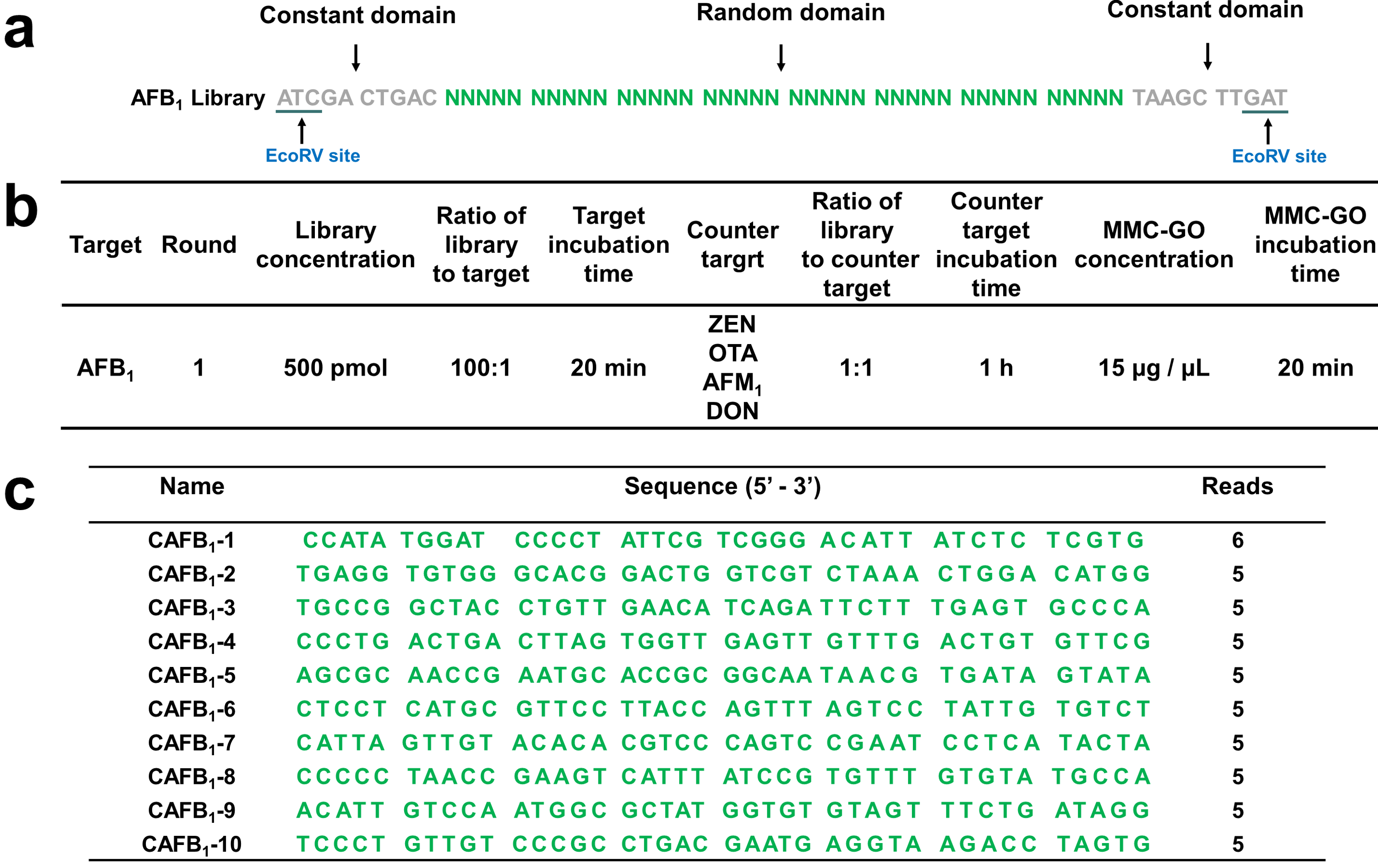

Supplement: Supplementary 1 — Supplementary Text Figs. S1 to S21 Table S1 and S2 [file research.0372.f1.zip › Figure S18.tif]

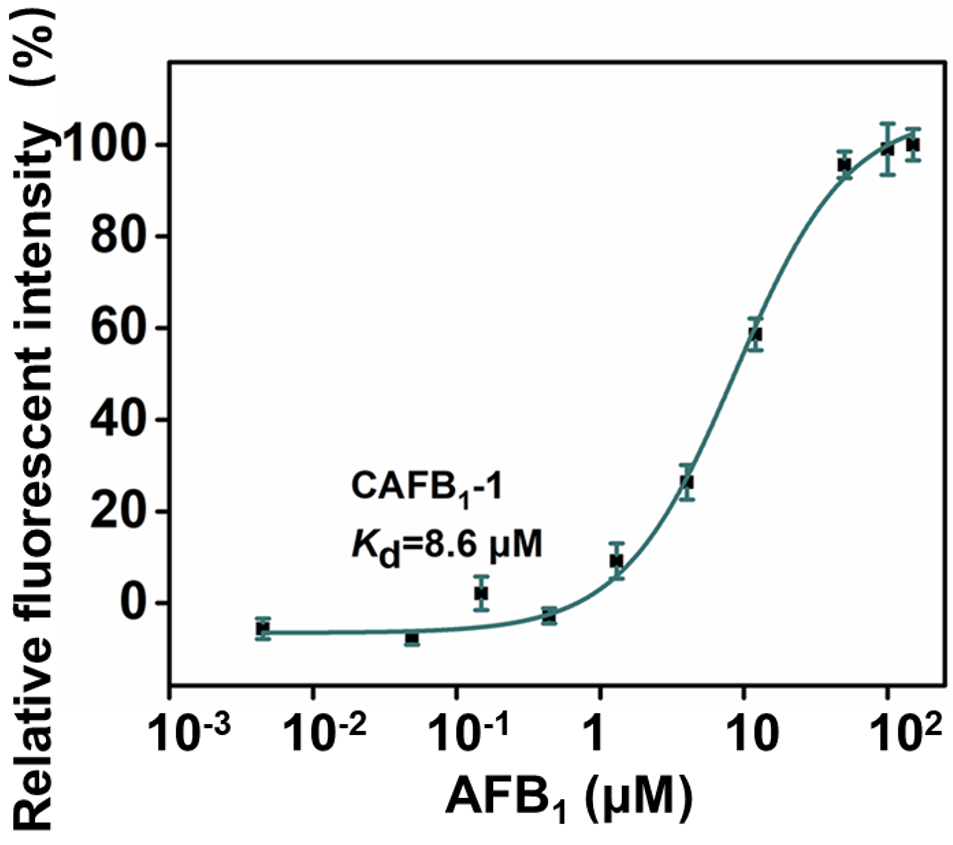

Supplement: Supplementary 1 — Supplementary Text Figs. S1 to S21 Table S1 and S2 [file research.0372.f1.zip › Figure S20.tif]

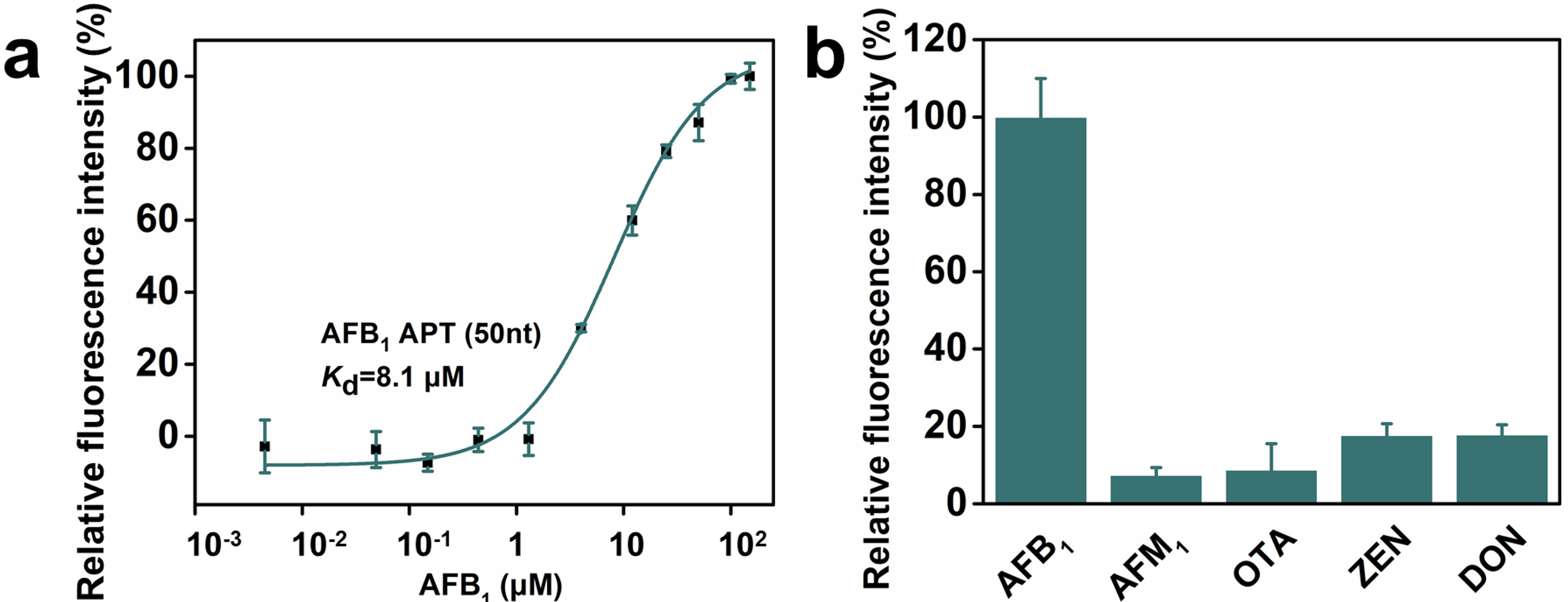

Supplement: Supplementary 1 — Supplementary Text Figs. S1 to S21 Table S1 and S2 [file research.0372.f1.zip › Figure S21.tif]

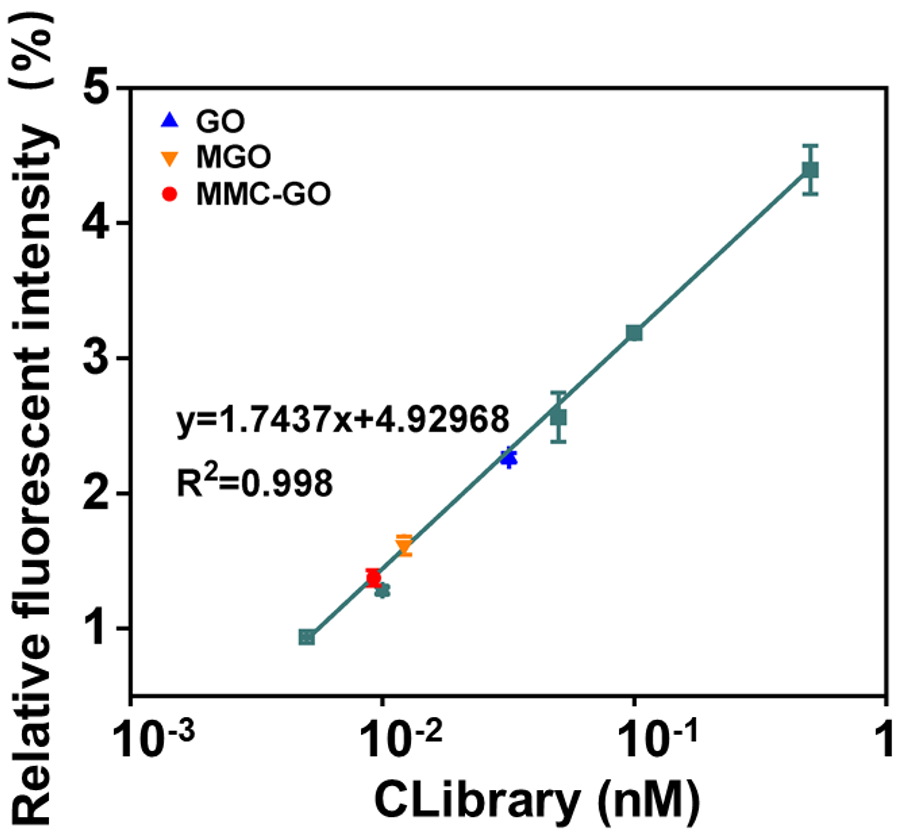

Supplement: Supplementary 1 — Supplementary Text Figs. S1 to S21 Table S1 and S2 [file research.0372.f1.zip › Figure S3.tif]

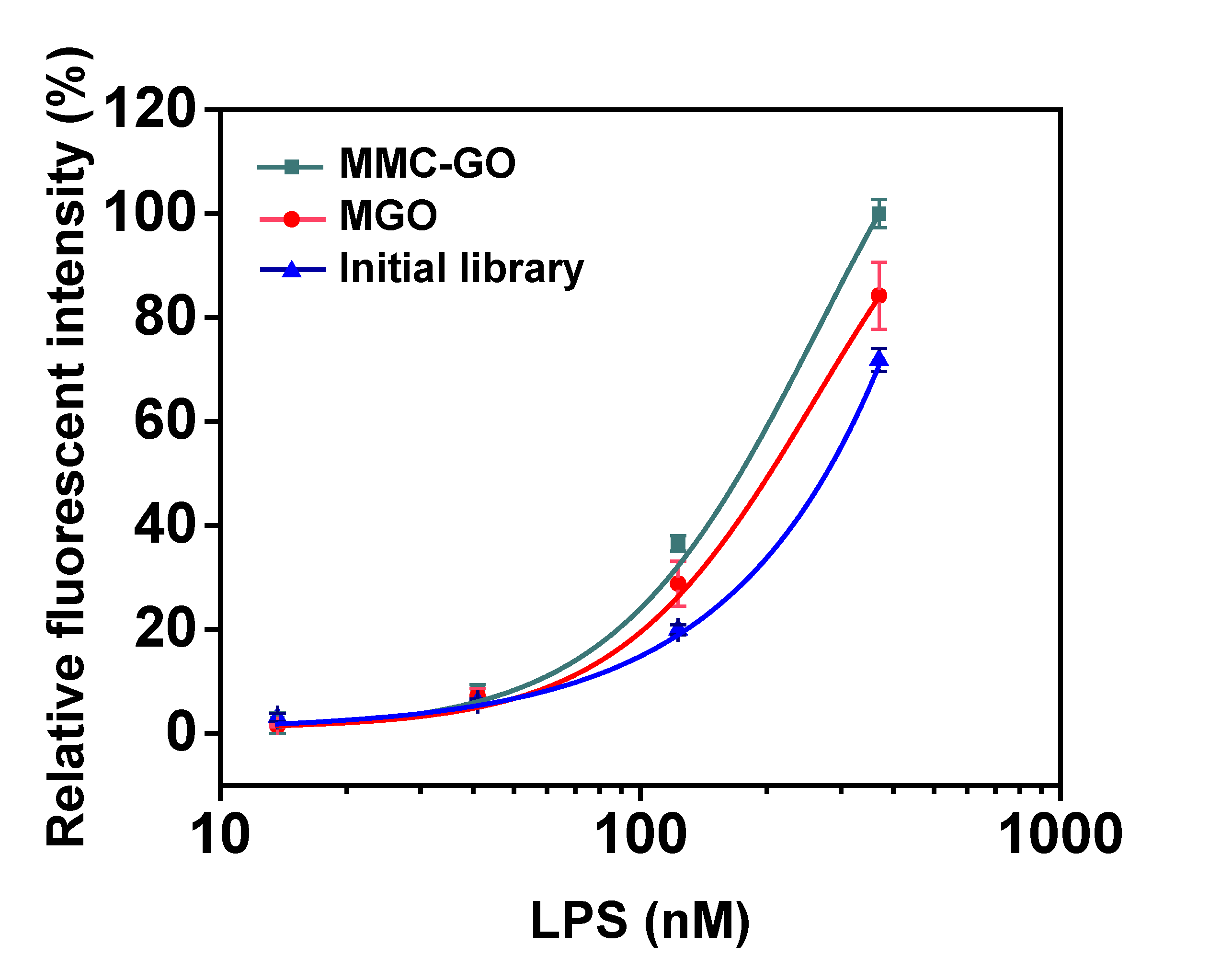

Supplement: Supplementary 1 — Supplementary Text Figs. S1 to S21 Table S1 and S2 [file research.0372.f1.zip › Figure S4.tif]

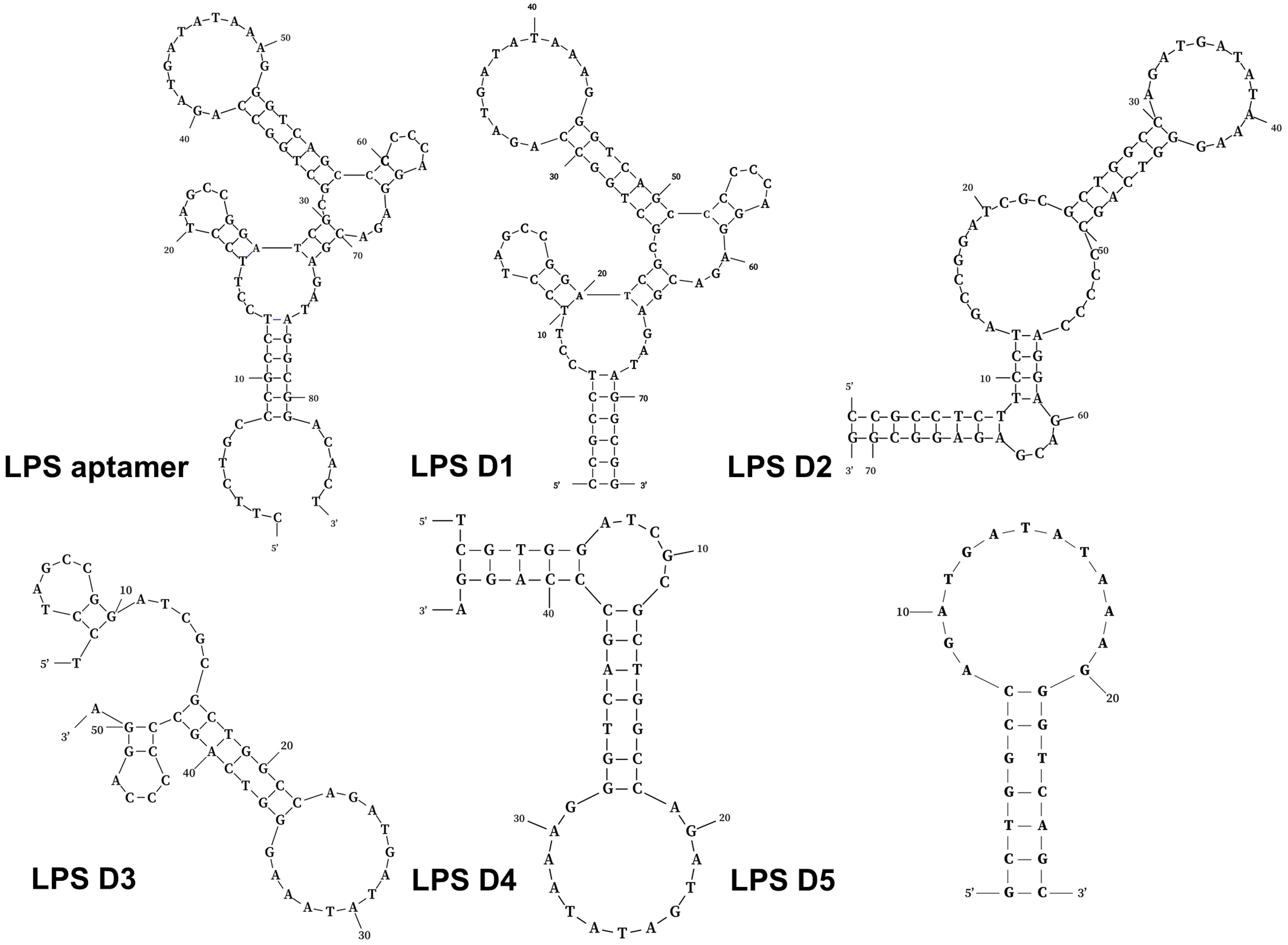

Supplement: Supplementary 1 — Supplementary Text Figs. S1 to S21 Table S1 and S2 [file research.0372.f1.zip › Figure S5.tif]

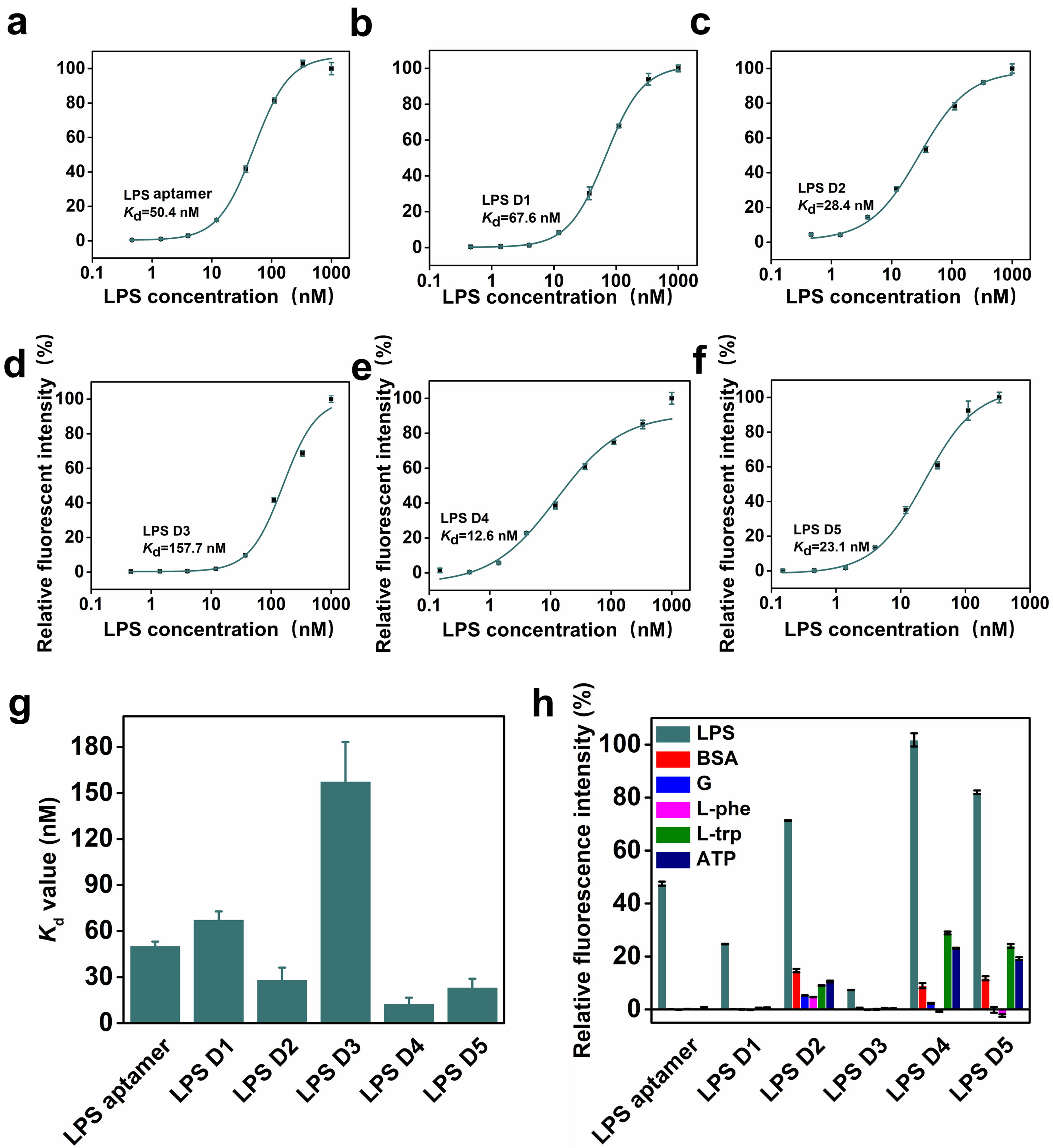

Supplement: Supplementary 1 — Supplementary Text Figs. S1 to S21 Table S1 and S2 [file research.0372.f1.zip › Figure S6.tif]

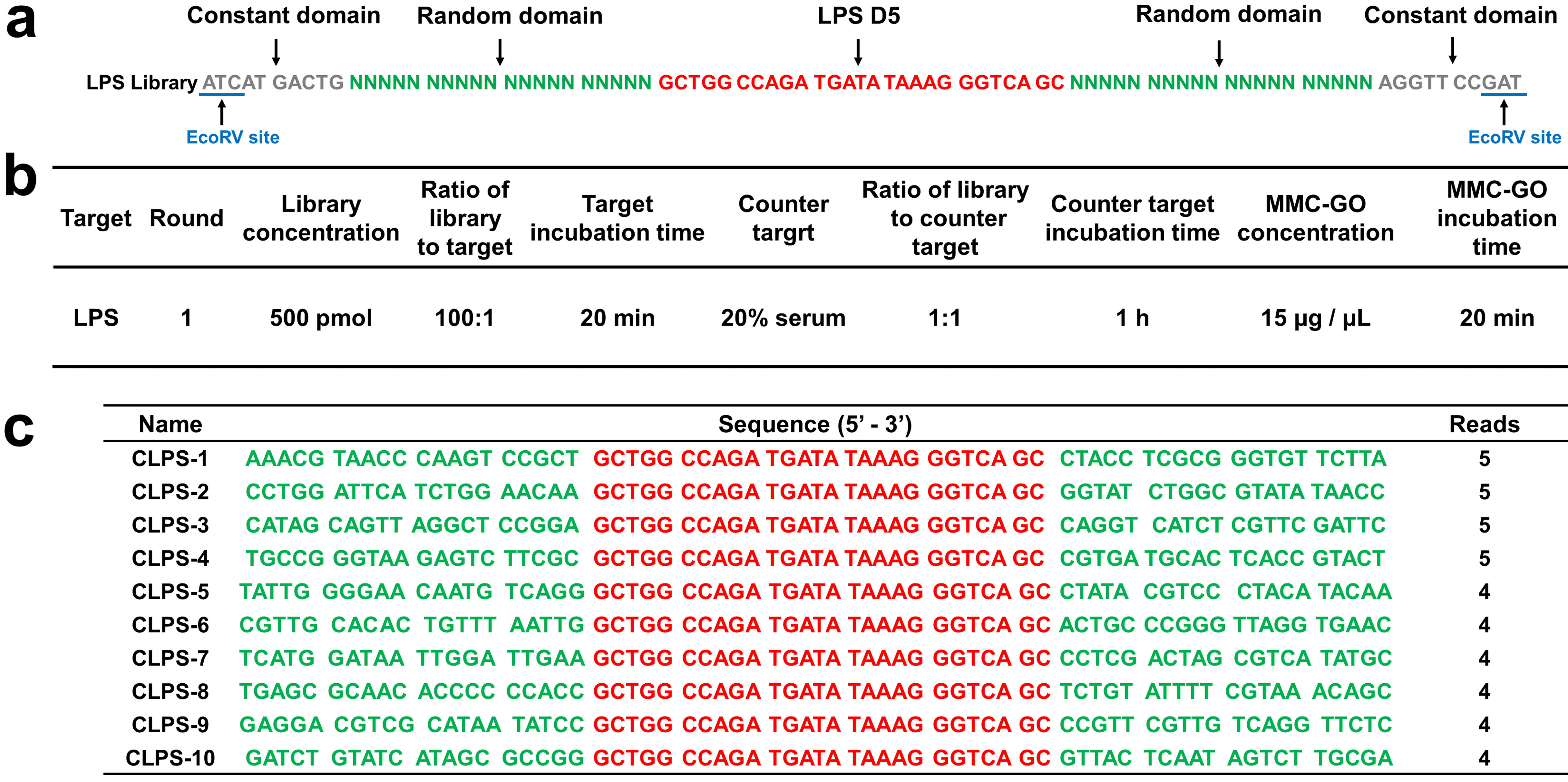

Supplement: Supplementary 1 — Supplementary Text Figs. S1 to S21 Table S1 and S2 [file research.0372.f1.zip › Figure S7.tif]

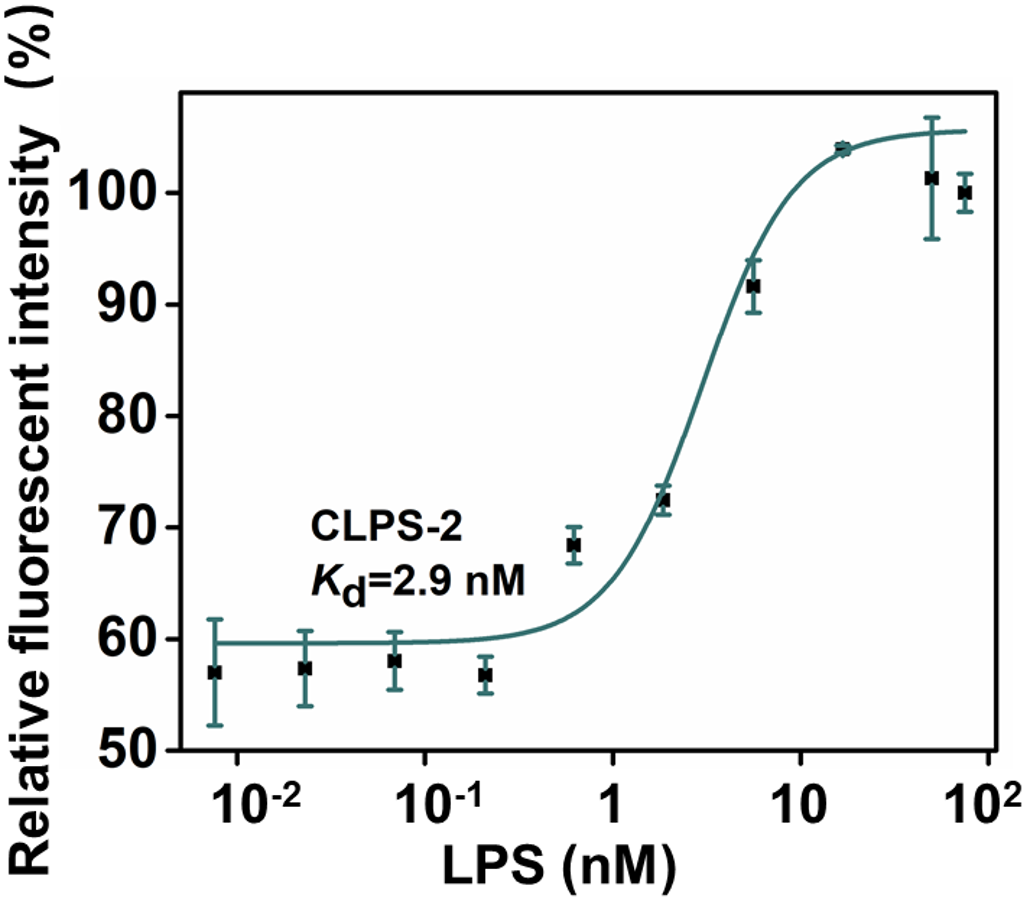

Supplement: Supplementary 1 — Supplementary Text Figs. S1 to S21 Table S1 and S2 [file research.0372.f1.zip › Figure S9.tif]
